# Turning cold tumors into hot tumors by improving T-cell infiltration

**DOI:** 10.7150/thno.58390

**Published:** 2021-03-11

**Authors:** Yuan-Tong Liu, Zhi-Jun Sun

**Affiliations:** 1 The State Key Laboratory Breeding Base of Basic Science of Stomatology (Hubei-MOST) & Key Laboratory of Oral Biomedicine, Ministry of Education, School and Hospital of Stomatology, Wuhan University, Wuhan, China.; 2 Department of Oral Maxillofacial-Head Neck Oncology, School and Hospital of Stomatology, Wuhan University, 237 Luoyu Road, Wuhan 430079, China.

**Keywords:** cold tumor, immune checkpoint inhibitors, T-cell infiltration, T-cell priming, nanomedicine

## Abstract

Immunotherapy, represented by immune checkpoint inhibitors (ICIs), has greatly improved the clinical efficacy of malignant tumor therapy. ICI-mediated antitumor responses depend on the infiltration of T cells capable of recognizing and killing tumor cells. ICIs are not effective in "cold tumors", which are characterized by the lack of T-cell infiltration. To realize the full potential of immunotherapy and solve this obstacle, it is essential to understand the drivers of T-cell infiltration into tumors. We present a critical review of our understanding of the mechanisms underlying “cold tumors”, including impaired T-cell priming and deficient T-cell homing to tumor beds. “Hot tumors” with significant T-cell infiltration are associated with better ICI efficacy. In this review, we summarize multiple strategies that promote the transformation of "cold tumors" into “hot tumors” and discuss the mechanisms by which these strategies lead to increased T-cell infiltration. Finally, we discuss the application of nanomaterials to tumor immunotherapy and provide an outlook on the future of this emerging field. The combination of nanomedicines and immunotherapy enhances cross-presentation of tumor antigens and promotes T-cell priming and infiltration. A deeper understanding of these mechanisms opens new possibilities for the development of multiple T cell-based combination therapies to improve ICI effectiveness.

## Introduction

Recently, immune checkpoint inhibitors (ICIs), such as nivolumab and pembrolizumab, have been applied to an increasing number of cancer types, forming a paradigm treatment in clinical trials 1, 2. Although ICIs have shown clinical activity in a wide range of tumor types, a substantial percentage of patients still do not respond to ICI therapy 3. ICI-mediated antitumor responses rely on the expression of PD-L1 in tumors and the infiltration of T cells capable of recognizing and killing tumor cells. Immune cells such as CD8^+^ T cells are associated with prolonged survival of cancer patients and increased efficacy of immunotherapy 4. A lack of T cells in tumors can lead to resistance to immunotherapy 5. The success of chimeric antigen receptor (CAR) T-cell infusions for patients' leukemia and lymphoma also demonstrates the importance of T cells in antitumor immunity 6. Considering the potential mechanisms of cancer immunotherapy, the infiltration of CD8^+^ T lymphocytes in tumors is important for the therapeutic response to ICIs.

**Figure 1:**
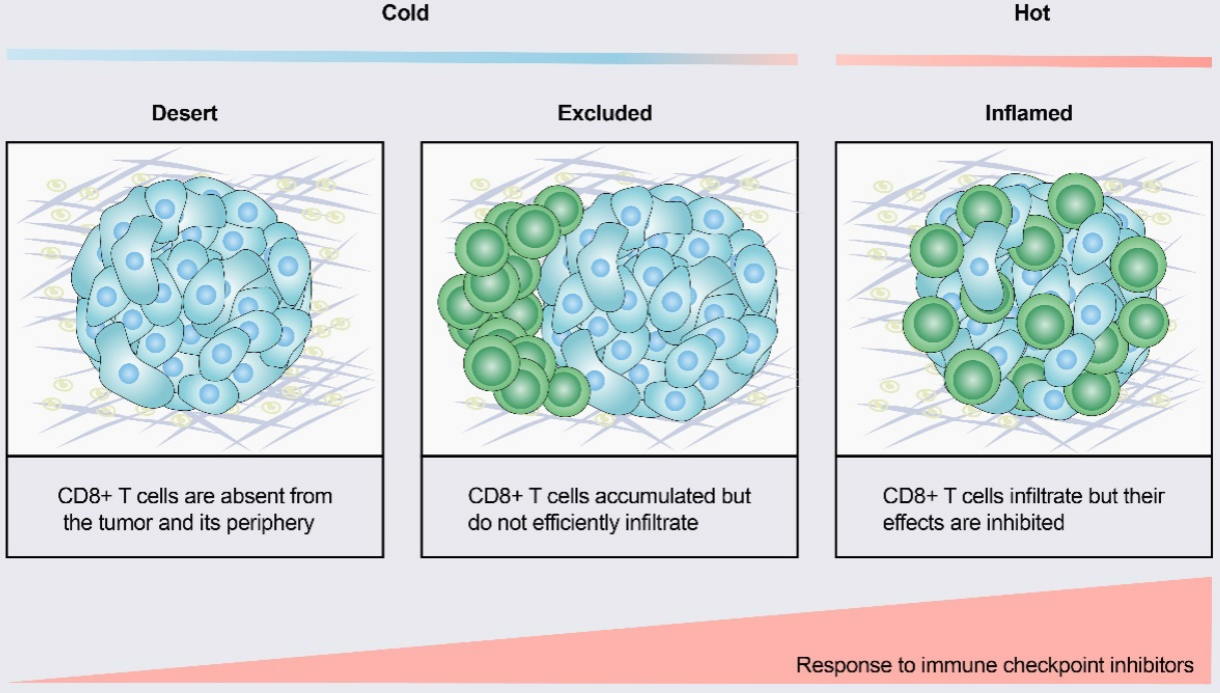
** Tumor immune phenotypes.** Based on the spatial distribution of CD8^+^ T lymphocytes in the tumor microenvironment (TME), a gradient of three immunophenotypes is observed: the immune-desert, immune-excluded and immune-inflamed phenotypes. In the immune-desert phenotype, immune cells are absent from the tumor and its periphery. In the immune-excluded phenotype, immune cells accumulate but do not efficiently infiltrate. In the immune-inflamed phenotype, immune cells infiltrate but their effects are inhibited. Notably, the three different phenotypes have different response rates to immune checkpoint inhibitors.

According to the spatial distribution of cytotoxic immune cells in the tumor microenvironment (TME), a tumor is classified into one of three basic immunophenotypes: immune-inflamed, immune-excluded and immune-desert phenotypes (Figure 1) 3. Immune-inflamed tumors, also named “hot tumors”, are characterized by high T-cell infiltration, increased interferon-γ (IFN-γ) signaling, expression of PD-L1 and high tumor mutational burden (TMB) 7. Tumors with an inflamed phenotype tend to be more responsive to ICIs 8, 9. Immune-excluded tumors and immune-desert tumors can be described as “cold tumors”. In immune-excluded tumors, CD8^+^ T lymphocytes localize only at invasion margins and do not efficiently infiltrate the tumor 10. In immune-desert tumors, CD8^+^ T lymphocytes are absent from the tumor and its periphery 10. In addition to poor T-cell infiltration, “cold tumors” are characterized by low mutational load, low major histocompatibility complex (MHC) class I expression and low PD-L1 expression 7. Immunosuppressive cell populations, including tumor-associated macrophages (TAMs) and T-regulatory cells (Tregs) and myeloid-derived suppressor cells (MDSCs), are also present in cold tumors 7. These features suggest that cold tumors lack innate immunity or that the innate antitumor immune features present in “cold tumors” may be ineffective due to the exclusion of immune cells 3. In contrast to the inflamed phenotype, cold tumors rarely respond to ICI monotherapy 9.

**Figure 2:**
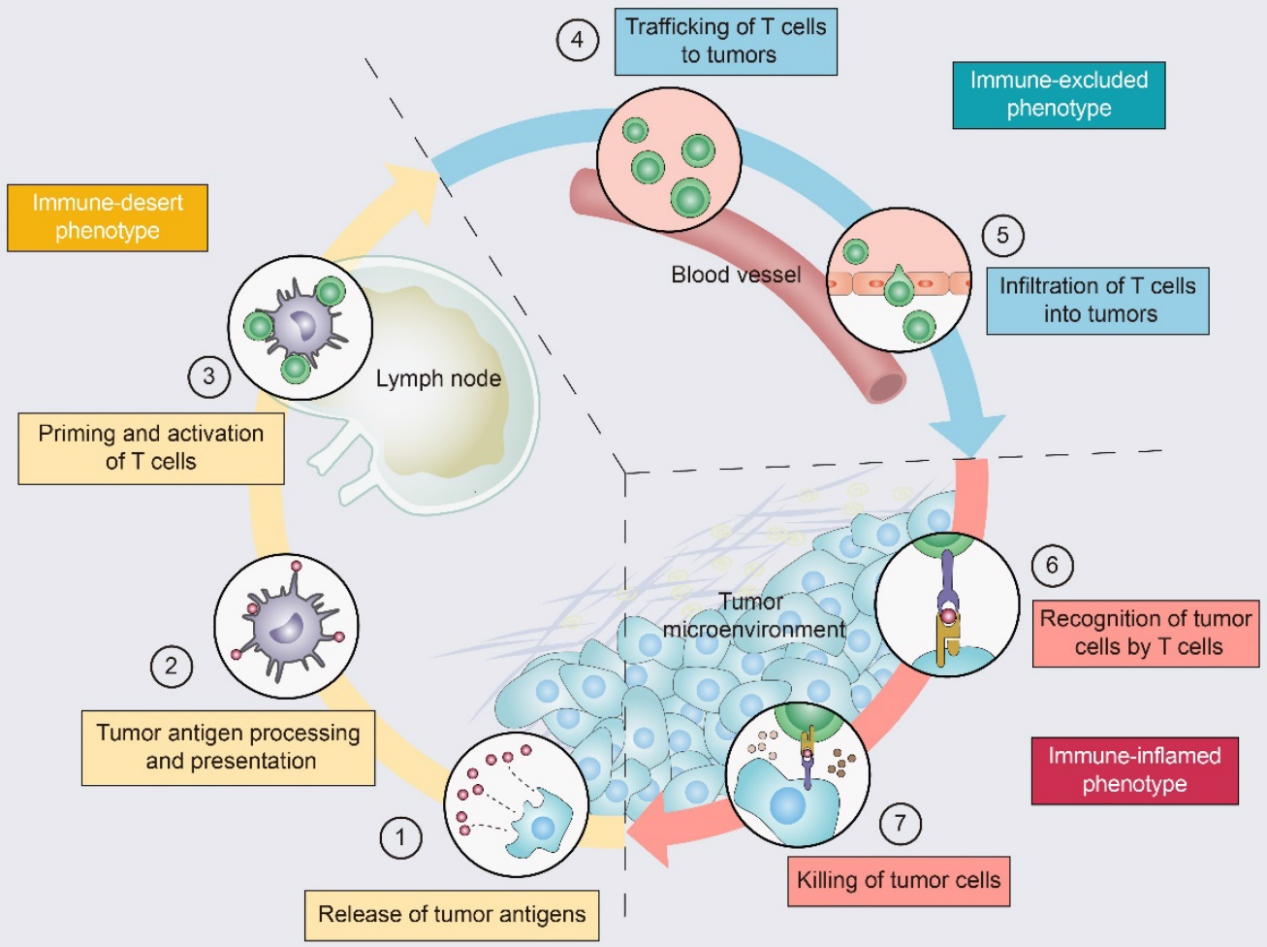
**The tumor-immunity cycle and three immunophenotypes.** Antitumor immunity is mediated to a large extent by CD8^+^ T lymphocytes. The tumor-immunity cycle consists of the following steps: **(1)** tumor antigen release, **(2)** tumor antigen processing and presentation, **(3)** T-cell priming and activation, **(4)** trafficking of T lymphocytes through the bloodstream to tumors, **(5)** infiltration of T lymphocytes into the tumor parenchyma from the vasculature or tumor periphery, **(6)** recognition of tumor cells, and **(7)** cytotoxic T lymphocyte (CTL) destruction of tumor cells by granule exocytosis or through the Fas/FasL pathway. Dead tumor cells release additional antigens, allowing the tumor-immunity cycle to continue. Notably, tumors with the immune-desert phenotype (yellow) cannot pass steps 1-3 due to the absence of T lymphocytes in both the tumor and its margins. Tumors with the immune-excluded phenotype (blue) cannot exceed steps 4-5 due to a lack of T lymphocytes in the tumor bed. Tumors with the immune-inflamed phenotype (red) cannot exceed steps 6-7 due to T-cell exhaustion and checkpoint activation. Adapted with permission from 11, copyright 2013 Elsevier.

Driving T cells into the TME is a gradual process (Figure 2): tumor cell death and antigen release, antigen-presenting cell (APC) processing and presentation of tumor antigens, and APC and T-cell interactions lead to T-cell priming and activation 11. Ideally, once activated, these tumor-specific T cells exit lymph nodes and travel through the bloodstream to tumor site 11. The production of T cells and their physical contact with tumor cells is crucial for the success of antitumor immunity 12. Once infiltrating the tumor bed, cytotoxic T lymphocytes (CTLs) specifically recognize antigenic peptide-MHC complexes on the surface of tumor cells, form immune synapses, and release perforin and granzyme to destroy the tumor cells 13. In addition, CTLs contribute to the apoptosis of tumor cells through the Fas/FasL pathway and suppress tumors by inducing ferroptosis and pyroptosis 14. Dead tumor cells release additional tumor antigens and thereby amplify the T-cell response 11.

With the development of nanotechnology, immunotherapy based on nanomedicines and biomaterials offers new opportunities for the future. Nanomedicines offer unique advantages in oncology treatment, such as improved drug precision and bioavailability and reduced immunotherapy-induced side effects 15. In addition, nanomedicines promote selective accumulation in tumors through enhanced permeability and retention (EPR) effects or include high-affinity ligands to achieve active targeting of tumors 15. Nanotechnology-based immunotherapy enhances tumor-specific immune responses, promotes the infiltration of CTLs, and inhibits tumor metastasis and recurrence.

Given the importance of T-cell infiltration, understanding the mechanisms of T-cell homing to the tumor is necessary. To improve the clinical benefit of immunotherapy, ICIs may be combined with strategies that convert “cold tumors” to “hot tumors”, which may make these tumors more sensitive to ICI therapy. In this review, we summarize the various mechanisms of T-cell infiltration disorders and current approaches to directing T cells into tumors. Finally, we summarize recent advances, challenges and opportunities for nanomedicine-based local therapeutic strategies to enhance T-cell infiltration and discuss further prospects in this field.

## Mechanisms of the “cold tumor” phenotype

**Figure 3:**
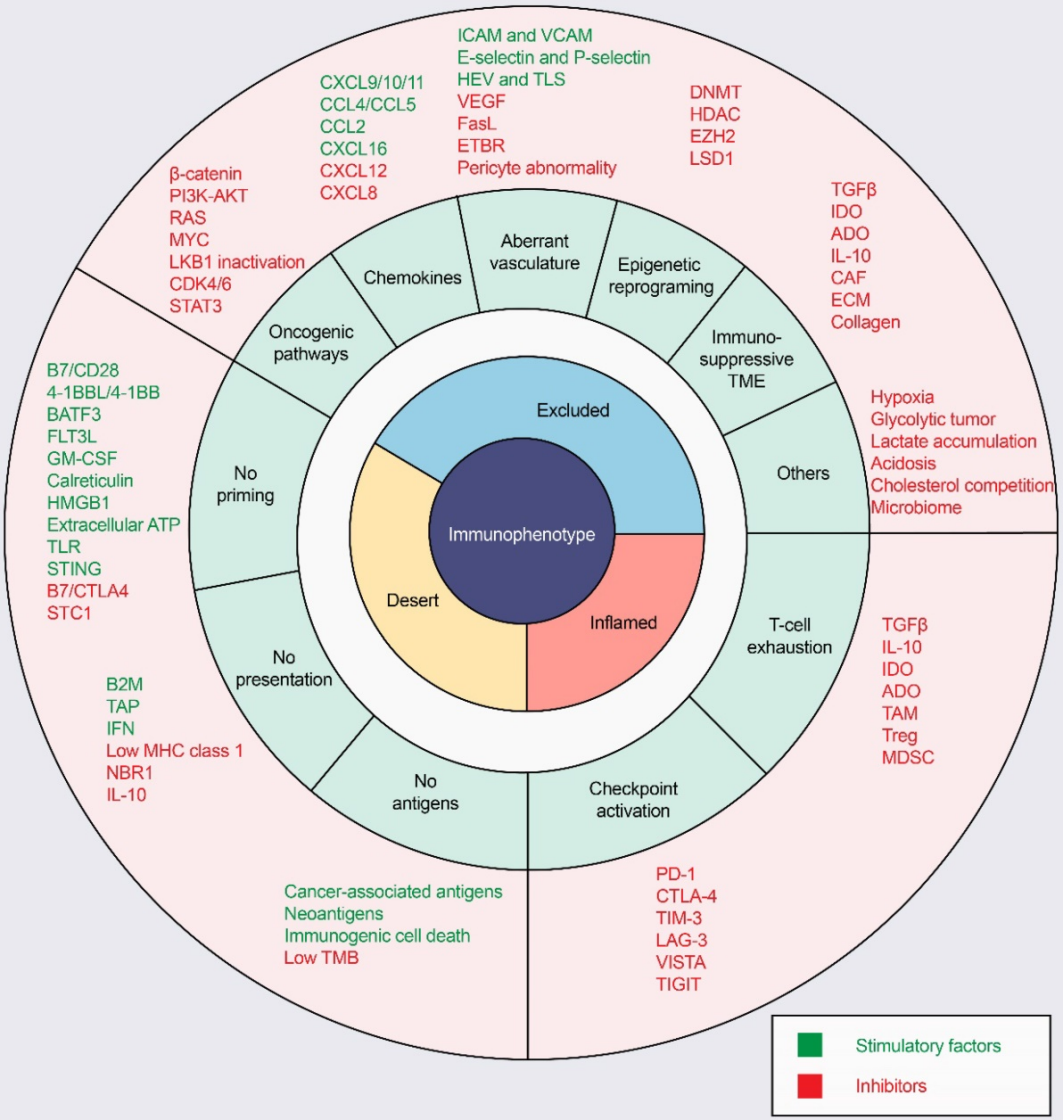
**Mechanisms of three distinct tumor phenotypes.** Three different phenotypes are associated with specific biological mechanisms. Tumors with the immune-desert phenotype (yellow) may lack T-cell priming due to the absence of tumor antigens, defective antigen processing and presentation machinery, or impaired DC-T-cell interactions. Tumors with the immune-excluded phenotype (blue) may exhibit activation of oncogenic pathways, aberrant chemokines, aberrant vasculature and hypoxia, or an immunosuppressive tumor microenvironment (e.g., stromal barriers). Tumors with the immune-inflamed phenotype (red) can be infiltrated by many immune cells, but these immune cells are suppressed due to checkpoint activation. ADO: adenosine; ATP, adenosine triphosphate; B2M: beta-2-microglobulin; BATF3: basic leucine zipper ATF-like transcription factor 3; CAFs: cancer-associated fibroblasts; CRT, calreticulin; CTLA4, cytotoxic T lymphocyte-associated antigen-4; CXCL: CXC-chemokine ligand; DNMT: DNA methyltransferase; ECM: extracellular matrix; ETBR: endothelin B receptor; EZH2: enhancer of zeste homolog 2; FLT3L: Fms-like tyrosine kinase 3 ligand; GM-CSF: granulocyte-macrophage colony-stimulating factor; HDAC: histone deacetylase; HEV: high endothelial venule; HMGB1: high mobility family protein B1; ICAM: intercellular adhesion molecule; IDO: Indoleamine 2,3-dioxygenase; IFN: interferon; IL: interleukin; MDSC: myeloid-derived suppressor cell; MHC: major histocompatibility complex; PD-1, programmed cell death protein 1; PD-L1, PD-1 ligand; STC1: stanniocalcin 1; TAM: tumor-associated macrophage; TAP: transporter associated with antigen processing; TGFβ: transforming growth factor-β; TIM3, T cell immunoglobulin and mucin domain-containing 3; TLR: Toll‑like receptor; TLS: tertiary lymphoid structure; TME: tumor microenvironment; Treg: T-regulatory cell; VCAM: vascular cell adhesion molecule; VEGF: vascular endothelial growth factor.

ICI response rates are low in “cold tumors”, as characterized by the absence of T-cell infiltration. In the process of driving T cells into tumors, there are many factors that can influence T-cell priming and T-cell homing to the tumor bed, leading to a noninflamed T-cell phenotype and failed antitumor immunity (Figure 3).

## Defective T-cell priming

### Lack of tumor antigens

The most direct cause of T-cell priming disorders is insufficient T-cell recognition due to a lack of tumor antigens. Table 1 summarizes the mechanisms of defects in T-cell priming. In general, targeted tumor antigens can be classified into two broad categories: nonmutated self-antigens and neoantigens generated by nonsynonymous somatic mutations 16. Self-antigens include nonmutated proteins that are aberrantly expressed or overexpressed in tumor cells, such as tumor-associated antigens (TAAs) and cancer/testis antigens (CTAs). Although self-antigens also elicit a tumor immune response, the primary target of the immune response is neoantigens, also termed tumor-specific antigens (TSAs). Neoantigens are specific to tumor cells and arise from somatic mutations in cancer genomes 16. The recognition of tumor neoantigens may promote T-cell priming and infiltration and can lead to a long-term clinical response 11, 16.

The TMB is broadly characterized as the number of total nonsynonymous single-nucleotide mutations in a tumor. In general, tumors with a higher TMB are believed to carry a higher neoantigen load that can be recognized by T cells, making them more likely to prime the immune system 17. Significant associations between high TMB and improved response to ICIs have been reported in a variety of tumor types 18, 19. The TMB has been used as a novel biomarker to predict the efficacy of programmed cell death protein 1 (PD-1) inhibitors 17, 19. Consistent with the importance of the efficacy of ICIs, a high TMB was associated with greater immune cell infiltration 20. Furthermore, a mutliomics network analysis revealed that in tumors characterized by recurrent mutations, such as melanoma and colorectal cancer, mutation or neoantigen burden was positively correlated with CTL infiltration 21. Considering the relationship between high TMB and tumor-specific T cells, low mutational load or neoantigen load contributes, in part, to the lack of immune infiltration and the acquisition of ICI resistance. However, in tumors characterized by recurrent copy number alterations, such as breast cancer, a correlation between tumor-specific T-cell infiltration and neoantigen load is lacking 21. An investigation of data on 266 melanomas in The Cancer Genome Atlas (TCGA) revealed no difference in antigen expression between cold and hot tumors 22. This finding suggests that other mechanisms, in addition to those involving low TMB, contribute to the absence of T-cell infiltration.

### Defects in tumor antigen processing and presentation machinery (APM)

After recognizing tumor antigens, APCs process the antigens and express the corresponding antigen peptide-MHC class I complex on its surface. However, alterations in the APM, such as downregulation of MHC-I molecule expression or the absence of beta-2-microglobulin (B2M), limit the presentation of antigen peptide-MHC class I complexes in the presence of tumor antigens. During antigen processing and presentation, transporters associated with antigen processing (TAP) transport cytosolic cleaved antigens to the endoplasmic reticulum for binding to the MHC. The deletion in TAP is related to defects in the antigen presentation process, which further affects the priming of T lymphocytes 23. B2M, the invariant chain of the MHC, is critical for the successful folding and transport of MHC-I to the cell surface 5. Knocking down the B2M gene in the M202 and M233 human melanoma cell lines resulted in the absence of MHC-I molecules expressed on their surface, and the absence of tumor-specific T-cell recognition and cytotoxicity 24. Identical results were observed in a B2M-knockout mouse model of lung cancer that showed resistance to PD-1 blockade 25. In addition, the lysosomal pathway has been linked to the reduced infiltration of CD8^+^ T lymphocytes. In pancreatic ductal adenocarcinoma (PDAC), the autophagy-associated receptor NBR1 induces the degradation of MHC-I on the cell surface of tumor cells, which in turn affects T-cell responses 26. These findings suggest that defects in tumor antigen processing and presentation pathways inhibit T-cell priming and the effectiveness of cancer immunotherapies.

### Dysfunctional DC-T cell interactions

Dendritic cells (DCs) are professional APCs with the unique ability to acquire antigens, migrate to secondary lymphoid organs (e.g., lymph nodes and spleen), and initiate the *in vivo* immune response. DC activation requires that pattern recognition receptors (PRRs) on their surface recognize “danger signals”, including pathogen-associated molecular patterns (PAMPs) and damage-associated molecular patterns (DAMPs) 27. This recognition enables DCs to present a tumor antigen peptide-MHC class I complex to T cells upon contact with them. DCs also express costimulatory signals such as B7 (including CD80 and CD86), providing the secondary signaling necessary for T-cell activation 28. Tumor cells can mediate diminished phagocytosis of DCs by trapping “danger signals”. For example, stanniocalcin 1 (STC1), an intracellular checkpoint, can trap DAMPs (e.g., calreticulin (CRT)) and inhibit DC phagocytosis and T-cell activation, contributing to tumor immune escape. Furthermore, STC1 is associated with low T-cell activation and poor survival in melanoma patients 29.

DCs are generally classified into two broad categories: plasmacytoid DCs (pDCs) characterized by the production of IFN-α, and conventional DCs (cDCs), which effectively stimulate T-cell proliferation 30. CDCs are further categorized into two distinct subsets: BATF3-dependent DCs and IRF4-dependent DCs 30. BATF3 DCs have the ability to cross-present tumor-derived antigens through the MHC-I pathway and thus initiate T cells 31. Furthermore, BATF3 DCs are the primary source of CXC-chemokine ligand 9 (CXCL9) and CXCL10, two key chemokines required to recruit CD8^+^ T cells expressing CXCR3 to tumors. There is a significant correlation between BATF3 DC markers (e.g., BATF3 and IRF8), the expression of CXCL9, CXCL10, and CXCL11 and the CD8^+^ effector T-cell phenotype in melanoma 22, 32. In the absence of BATF3 DCs, CD8^+^ effector T cells fail to migrate to tumors and antitumor immunity is thus defective 32. This finding validates the notion that BATF3 DCs may be essential for the priming and recruitment of the endogenous T cells necessary to counteract tumors.

The regulation of Fms-like tyrosine kinase 3 ligand (FLT3L) and granulocyte-macrophage colony-stimulating factor (GM-CSF) is important for the differentiation and recruitment of DCs 33. FLT3L is a growth factor that promotes the differentiation of hematopoietic progenitor cells from the bone marrow to the DC lineage 33. Tumor-derived FLT3L increased the infiltration of BATF3 DCs and CD8^+^ T lymphocytes in mouse tumors and enhanced migratory and resident DC subsets in draining lymph nodes (DLNs), suggesting a mobilizing effect of FLT3L on DC cells 34. Deficiency of FLT3L or GM-CSF resulted in a reduced number of DCs in secondary lymphoid organs and attenuated T-cell immune responses 35. Given the important role of DC-T cell crosstalk in naïve T-cell priming, impaired DC activation, a lack of DCs, and the overexpression of cosuppressive signals can lead to impaired T-cell activation.

## Deficient T-cell homing to the tumor bed

### Oncogenic pathway activation

For the purposes of this review, we summarize the mechanisms by which T cells are prevented from homing to the tumor bed (Table 2). There is growing evidence showing that the activation of tumor cell oncogenic pathways is related to the “cold tumor” phenotype and the potential for immunotherapy resistance. In WNT/βcatenin-positive melanoma tumors, reduced production of CCL4 results in decreased recruitment of BATF3 DCs to the TME 36. Ultimately, in the absence of the CXCL9 and CXCL10 produced by BATF3 DCs, CTLs are not recruited to the tumor 32. An analysis of human metastatic melanoma samples showed a negative correlation between CD8A expression and activation of the β-catenin signaling pathway 36. Direct injection of BATF3 DCs helped restore T-cell infiltration in β-catenin-positive tumors and resulted in modest tumor suppression 36. This outcome suggests that WNT/β-catenin signaling activation and defective BATF3 DC recruitment mediate T-cell exclusion and tumor cell escape from the immune system. Notably, activation of this oncogenic pathway excludes CTLs only when β-catenin is located into the nucleus 37. This finding indicates that the exclusion mechanism of CTLs is related to a transcriptional program specifically induced by β-catenin. In addition, only 48% of “cold” melanomas show active βcatenin signaling, suggesting that other oncogenic pathways may mediate immune exclusion 36.

Loss of PTEN activates the PI3K/AKT pathway, which is related to a noninflamed T-cell phenotype and immune resistance of melanoma. Loss of PTEN expression has been found to reduce the lipidation of the autophagosome protein LC3, resulting in decreased autophagic activity, which inhibits T-cell priming and the T-cell-mediated antitumor response 38. CD8^+^ T-cell infiltration in PTEN-deficient melanoma was significantly reduced compared to that in PTEN-expressing tumors. The results from a TCGA dataset analysis indicated that the expression of T-cell effector molecules (e.g., IFN-γ and granzyme B) was significantly reduced in melanomas with low PTEN expression 38.

As the gene with the most common mutations associated with cancer progression, RAS can lead to the activation of multiple signaling pathways, such as MAPK and PI3K, driving tumorigenesis 39. In addition, oncogenic K-RAS mutations mediate inflammation and crosstalk with the TME. For example, oncogenic K-RAS mutations induce tumor-promoting inflammation through the production of inhibitory cytokines (e.g., IL-6 and IL-8), the activation of NLRP3 inflammasome, and the release of chemokines (e.g., CCL5 and CCL9) 39.

Furthermore, oncogenic signaling through MYC enhances the expression of CD47 and PD-L1 on tumor cells. CD47 binds to inhibitory receptor signal-regulated protein-α (SIRPα) on the surface of APCs such as macrophages and DCs, which can prevent phagocytosis of tumor cells and interfere with antigen uptake 31, 40. Oncogenic KRAS and MYC synergistically induce immune regulation. For example, co-activation of KRAS and MYC in a mouse lung cancer model leads to the production of CCL9 and IL-23. This mediates stromal reprogramming, promotes angiogenesis, and excludes T and B cells and NK cells from tumors 41. It has also been found that inactivating mutations of LKB1 in non-small cell lung cancer (NSCLC) are related to increased neutrophil and decreased T-cell infiltration in a preclinical mouse model 42. In addition, CDK4/6 and STAT3 activation is associated with a noninflamed T-cell phenotype 43-45. Taken together, these results reveal that the activation of oncogenic pathways can affect not only tumor cells but also T cell-mediated antitumor immunity.

### Chemokines and their epigenetic regulation

The interaction between some chemokine receptors on effector T lymphocytes and corresponding chemokines may affect the trafficking of effector T lymphocytes to tumor sites. The lack of several chemokines, including CXCL9, CXCL10, CCL4, CCL5, CXCL16 or CX3CL1, has been reported to lead to T-cell exclusion 46, 47. Considering the importance of the TH1-type chemokines CXCL9 and CXCL10 to T-cell recruitment, certain tumors show low levels of CXCL9 and CXCL10 expression, which may explain the reduced infiltration of effector T lymphocytes into these tumor beds 32, 36. For example, BATF3 DCs are the major sources of CXCL9 and CXCL10, and a lack of BATF3 DCs leads to low expression of CXCL9 and CXCL10. In addition, epigenetic regulation in tumors is also important for maintaining low expression levels of these cytokines. DNA methyltransferase (DNMT) and enhancer of zeste homolog 2 (EZH2) can mediate DNA methylation and histone lysine methylation, respectively, to suppress the expression of CXCL9 and CXCL10 in ovarian cancer 48. Similar results have been confirmed in colon cancer 49. In preclinical models, treatment with epigenetic modulators promoted tumor infiltration of effector T cells and enhanced the effect of anti-PD-L1 48. In addition to CXCL9 and CXCL10, CCL5 expression is positively related to CD8^+^ T-cell infiltration 21, 47. The binding of CCL5 to CCR5 promotes the recruitment of CD8^+^ T cells. However, DNA methylation leads to deletion of CCL5 expression, which in turn contributes to the absence of CD8^+^ T-cell infiltration 50. In mouse models of NSCLC, the combined use of DNMT inhibitors and histone deacetylase (HDAC) inhibitors increased the expression of endogenous retrovirus (ERV), which in turn induced type I IFN responses. This combination treatment reversed the immune resistance of NSCLC models by downregulating oncogenic MYC signaling, leading to an increase in CCL5 and promoting T-cell infiltration into tumors 51.

However, some chemokines are detrimental to the trafficking of T cells to tumor beds. Stromal cells, especially cancer-associated fibroblasts (CAFs), are the main producers of CXCL12. CXCL12 produced by CAFs misdirects CTLs to the extratumoral stroma and prevents CTLs from entering the tumor 52. Furthermore, elevated CXCL8 expression has been reported to be associated with a reduction in the number of T cells in tumors, increased neutrophil and monocyte infiltration, and limited responses to ICIs 53, 54. These results reveal the regulatory effect of chemokine receptor and ligand interactions on CTL homing to tumors and their integration into the TME.

### Aberrant vasculature and hypoxia

Adequate T-cell infiltration in the tumor bed is not only dependent on the recruitment of the appropriate chemokines but is also controlled by the tumor vasculature. During the trafficking of CD8^+^ T lymphocytes to a tumor, they must enter the tumor circulatory system, adhere to vascular endothelial cells and migrate across the vessel wall 11. The recruitment of CD8^+^ T cells to tumors requires the action of vascular endothelial adhesion molecules, including P-and E-selectin, intercellular adhesion molecules (ICAMs), and vascular cell adhesion molecules (VCAMs) 55, 56. However, the downregulation or ineffective aggregation of adhesion molecules on tumor endothelial cells leads to endothelial cell anergy and reduced effector T-cell trafficking to tumor sites 55, 57. Endothelin binds to a corresponding receptor on endothelial cells, endothelin B receptor (ETBR), and reduces ICAM-1 production, thereby inhibiting CD8^+^ T cell adhesion to endothelial cells 58. Additionally, vascular endothelial growth factor (VEGF), produced by tumor and stromal cells, stimulates the proliferation of endothelial cells, leading to new vessel formation, often accompanied by impaired tissue perfusion and increased vascular permeability 59. VEGF also decreases the expression of important molecules, such as VCAM-1, on the cell surface of the endothelium, ultimately preventing T cells from migrating to the TME 59. Another mechanism through which tumor endothelial cells can inhibit T-cell migration is modulation of immune cell activity or viability. IL-10, prostaglandin E2 (PGE2), and VEGF induced FasL upregulation in tumor endothelial cells to kill tumor-associated T cells, and anti-FasL attenuated this killing effect. Acetylsalicylic acid (ASA), which inhibits COX and PGE2 activity, and anti-VEGF antibodies promoted CD8^+^ T lymphocyte infiltration in the TME and improved prognoses 60. Furthermore, pericyte abnormalities and inadequate coverage prevent the maintenance of endothelial cell integrity, resulting in the dysfunctional leakage and flow characteristic of tumor vasculature 56, 61. However, other structures can promote the translocation of CD8^+^ T lymphocytes from blood vessels to tumor sites. The formation of high endothelial venules (HEVs) and related tertiary lymphoid structures (TLSs) facilitates T-cell migration to the TME and is often associated with better prognoses 46, 62.

In addition, impaired vascular tight junctions and increased permeability result in the promotion of hypoxia, acidosis and necrosis, which inhibit immune effector T-cell functions and antitumor immunity 56. As a hallmark of cancer, hypoxia is caused by increased oxygen demand due to tumor cell proliferation and inadequate blood supply due to angiogenesis 63. Hypoxia-inducible factor 1 (HIF1) is a key transcription factor activated by hypoxia 64. Hypoxia inhibits T-cell infiltration in several ways. First, hypoxia promotes the recruitment of immunosuppressive cells to the TME 65. Second, hypoxia-induced CCL28 and VEGF promote angiogenesis and affect T-cell trafficking 56, 66. Finally, the expression of two ectonucleotidases, CD39 and CD73, can be upregulated in tumors in response to hypoxia and transforming growth factor-β (TGFβ) 67. CD39 and CD73 catalyze the sequential conversion of ATP to extracellular adenosine (ADO) 68. ADO binds to the adenosine A2A receptor (A2AR) and inhibits the production of cytokines such as IL-2 and the development and proliferation of T cells 69. The inhibition of A2AR increased T-lymphocyte infiltration and led to improved tumor control in mouse melanoma models, suggesting a potential effect of the ADO signaling pathway in promoting T-cell exclusion 70. In addition, ADO can weaken antitumor immunity by inhibiting the effector functions of NK cells and DCs and by promoting the recruitment and polarization of MDSCs and Tregs 71.

### The TME: immunosuppressive cells and factors

The immunosuppressive microenvironment at tumor sites, including dense stroma and immunosuppressive cells and factors, can prevent T-cell priming and infiltration in “cold tumors”. TGFβ is a potent immunosuppressive cytokine that promotes immune escape and blocks the acquisition of the TH1-effector phenotype 72. CAFs, which are abundant in the TME, are the main producers of TGFβ. Increased TGFβ production by CAFs is associated with T-cell exclusion from the tumor and a poor response to atezolizumab 73. TGFβ limits the proliferation of CD4^+^ T lymphocytes by inhibiting the production of IL-2 and induces the conversion of naïve CD4^+^ T lymphocytes into Tregs 74, 75. TGFβ also negatively affects DC differentiation and antigen-presenting functions, which interfere with T-cell priming 76. In summary, TGFβ hinders antitumor immunity by affecting T-cell differentiation and function and preventing T-cell infiltration into tumors.

Tryptophan metabolism is often dysregulated in a broad range of cancers and is associated with immune resistance. Indoleamine 2,3-dioxygenase (IDO) in tumor cells converts the essential amino acid tryptophan into kynurenine, which blocks the priming of T lymphocytes and facilitates the development of Tregs 77. IDO also recruits and activates MDSCs and inhibits the accumulation of tumor-specific T lymphocytes in tumors 78. IDO inhibitors such as epacadostat and navoximod have been used in combination with ICIs with promising results in clinical trials 79. However, the failure of epacadostat in combination with pembrolizumab in the phase III clinical ECHO-301 study indicates that the effectiveness of drugs targeting IDO needs to be further considered 80.

CAFs are key cellular components in the tumor stroma and can promote tumor growth 81. CAFs are predominantly located at the infiltrating edges of tumors, regulating tumor metastasis and influencing angiogenesis by synthesizing and remodeling the extracellular matrix (ECM) and producing cytokines, and transforming tumor margins into immune “cold” zones 52, 82. CAFs led to immunosuppression and T-cell exclusion through several mechanisms. First, CAFs produce extracellular matrix that forms a physical barrier to prevent T-cell infiltration into the tumor area 83. Second, CXCL12 produced by CAFs has been shown to inhibit T-lymphocyte infiltration within tumors in a pancreatic cancer model 84. Third, CAFs can also reduce T-cell responses and exert immunosuppressive effects through the production of TGFβ and IL-6 82. Reprogramming CAFs is an effective strategy to “normalize” the TME. This strategy reduces ECM levels, decompresses blood vessels, and increases the degree of T-cell penetration to improve cancer treatment 85.

In addition, TAMs exclude T cells from the tumor by regulating the ECM and mediating the nitration of CCL2 and CCL5 86, 87. TAMs affect T-cell recruitment by promoting abnormal angiogenesis through the production of VEGF and matrix metalloproteinase-9 (MMP9) 88. Cytokine colony-stimulating factor-1 (CSF-1) and CSF-1R interactions are capable of promoting myeloid cell differentiation towards an immunosuppressive M2 macrophage phenotype. Targeting TAMs with CSF1R inhibitors reduces the number of TAMs and increases the infiltration of effector lymphocytes such as CD8^+^ T cells 89.

Tumor cells are typically characterized by a high rate of glucose uptake and active glycolysis, even in the presence of oxygen. This phenomenon is known as the “Warburg effect”. In this process, glucose is rapidly consumed and the abundance of lactate in the TME increases. The glucose-deficient, lactate-rich TME exerts metabolic stress on infiltrating T cells, leading to local immunosuppression and ICI resistance 77. Glucose deprivation in the TME metabolically mediates T cell hyporeactivity, inhibits mTOR activation, and reduces glycolytic capacity and IFN-γ production 90. In addition, glycolytic activity and T-cell infiltration are negatively correlated in a variety of tumors 91-93. Consistent with this observation, high glucose-transporter 1 (GLUT-1) expression in renal cell carcinoma are associated with low infiltration of CD8^+^ T cells 92. These results suggest an association of glycolytic (Warburg) tumors with a noninflamed T-cell phenotype. Interestingly, in addition to tumor cells, stromal cells, such as CAF and TAM, can also promote lactate accumulation in the TME through the so-called “Reverse Warburg effect” 91. Targeting glucose metabolism and lactate production in tumor and stromal cells, such as inhibition of LDH-A, may be an effective strategy to promote T-cell infiltration 94. Lactate accumulation and acidification of the TME suppress antitumor immunity. Lactate-induced acidosis impairs the differentiation of monocytes to DCs and inhibits the antigen-presenting function of DCs, which in turn inhibits T-cell activation 95. High concentrations of lactate and acidification in the TME inhibit monocarboxylate transporter 1 (MCT1)-mediated lactate release from T cells and suppress the proliferation of T cells that utilize aerobic glycolysis 96. In addition, lactate inhibits the chemotaxis and antitumor activity of CTLs and promotes tumor immune escape 96. Inhibition of lactate production or restoration of physiological pH of the TME can reverse the inhibitory effect of lactate on antitumor immunity. For example, neutralizing tumor acidity with sodium bicarbonate in combination with ICIs or adoptive cellular therapy (ACT) can effectively promote T-cell infiltration and improve antitumor responses in a variety of mouse tumor models 97.

In addition to glucose, metabolic competition between tumors and immune cells includes amino acids and fatty acids. For example, the high rate of cholesterol esterification in tumors inhibits T cell receptor (TCR) aggregation and immune synapse formation 77. The cholesterol esterification key enzyme ACAT1 inhibitor avasimibe can promote the proliferation of CD8^+^ T cells and exhibit good antitumor effects 98. New studies have also confirmed that inhibition of PCSK9, a key protein regulating cholesterol metabolism, upregulates MHC-I levels on the surface of tumor cells, increases intratumoral infiltration of CTLs, and synergistically inhibits tumor growth with anti-PD1 antibodies 99. Considering the interaction between tumor metabolism and immune cell metabolism, navigating metabolic pathways to reduce metabolic stress on T cells is a promising strategy to improve the efficacy of immunotherapy.

## Therapeutic approaches to drive T cells into tumors

**Figure 4:**
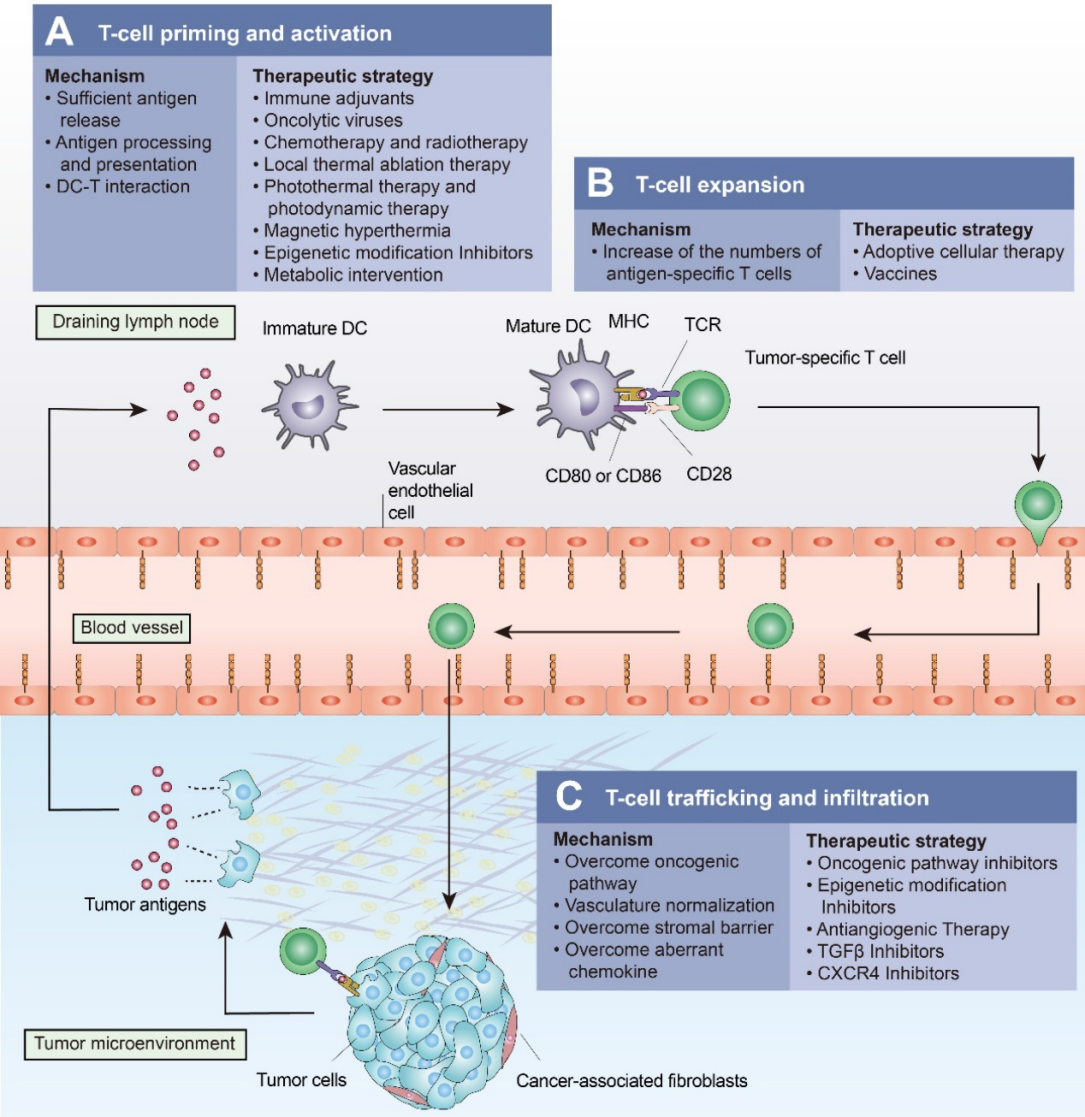
**Approaches to turn a “cold tumor” into a “hot tumor”.** Some representative approaches that lead to increased T-cell infiltration and improved efficacy of immune checkpoint inhibitors are highlighted here. **(A)** Oncolytic viruses, local thermal ablation therapy (e.g., radiofrequency ablation), chemotherapy, and radiotherapy are all capable of inducing immunogenic cell death (ICD) to promote T-cell priming and activation. Local administration of immune adjuvants such as TLR agonists promotes the activation of dendritic cells (DCs). Epigenetic modification inhibitors can promote T-cell priming by increasing the expression of tumor antigens and by restoring antigen processing and presentation mechanisms. **(B)** Cancer vaccines and adoptive cellular therapies, such as CAR-T cells, can promote the expansion of tumor-specific T lymphocytes. **(C)** Intrinsic oncogenic pathway inhibitors, epigenetic modification inhibitors, antiangiogenic therapies, TGFβ inhibitors, and CXCR4 inhibitors promote T-cell trafficking and enable T cells to infiltrate the tumor more effectively.

ICIs have revolutionized cancer treatment by activating T-cell-based antitumor immunity. However, a significant number of patients show a poor response to ICIs due to the multiple mechanisms mediating T-cell exclusion. Several approaches have been shown to drive T cells into tumors. These approaches “fire up” “cold tumors” to improve the efficacy of ICIs (Figure 4 and Table 3).

## Therapeutic approaches to promote T-cell priming

### Immune adjuvants

Innate immune sensing pathways play critical roles in the development of antitumor immunity. The PRR family includes Toll-like receptors (TLRs), NOD-like receptors (NLRs), RIG-I-like receptors (RLRs), and C-type lectin receptors (CLRs) 27. When TLRs are stimulated, DCs can produce a variety of proinflammatory cytokines, including tumor necrosis factor (TNF), IL-1, and type I IFNs 27. DCs are the main sources of type I IFNs, which facilitate the expression of MHC-I on the surface of tumor cells and the maturation of DCs, thereby promoting T-cell priming 100.

In contrast to therapies such as vaccines and CAR-T cells, immune adjuvants harness the endogenous antigen repertoire in the tumor and have been used to enhance the immune response for the treatment of malignant tumors. Local administration of the TLR7/8 agonist imidazoquinoline with coupled nanoparticles significantly activated the DCs of secondary lymphoid organs, upregulated the expression of MHC-II, CD40, and CD86 on their surface, and expanded the number of tumor-specific CD8^+^ T lymphocytes, which inhibited tumor growth 101. In clinical trials of patients with advanced malignant melanoma, combined treatment with the TLR9 agonists SD-101 and pembrolizumab resulted in increased type I IFN production and CD8^+^ T-cell infiltration and potentially improved clinical efficacy 102.

As DNA receptors in the cytoplasm, cyclic GMP-AMP synthase (cGAS) in DCs, macrophages, and other immune cells recognizes aberrant DNA in the cytoplasm and catalyzes the formation of cGAMP, which subsequently activates the STING signaling pathway 103. Activation of the STING signaling pathway mediates the expression of proinflammatory cytokines (e.g., type I IFNs) and chemokines (e.g., CXCL10) in a TBK1-IRF3-dependent manner, thereby initiating the antitumor immune response 104. The systemic cGAMP mimetic SR-717 activated the STING signaling pathway, promoted CD8^+^ T-cell, NK cell, and CD8α^+^ DC activation, and significantly inhibited tumor growth 105. SR-717 also induced PD-L1 expression in a STING-dependent manner, revealing the significance of the combination of STING agonists and ICIs for tumor treatment. In mouse tumor models with a low response to PD-1 blockade, the combination of PD-1 blockade and the STING agonist MSA-2 increased the infiltration of tumor CD8^+^ T lymphocytes and better inhibited tumor growth 106.

### Oncolytic viruses (OVs)

OVs are now being recognized as emerging therapeutics with potent anticancer activity. In addition to selective tumor lysis, they can activate both innate and adaptive immune responses, resulting in alterations in the TME. First, lysis of tumor cells by OVs induces immunogenic cell death (ICD), leading to a massive release of intracellular TAAs, PAMPs, and DAMPs 107. Three DAMPs are released during ICD: passively released high mobility family protein B1 (HMGB1), actively secreted extracellular ATP, and cell surface-expressed CRT 108. These DAMPs act as adjuvants to promote DC uptake and cross-present tumor antigens to T lymphocytes in DLNs. OVs also improve the function of DCs by stimulating their production of type I IFNs. Immune adjuvants interact with tumor antigens in tumor residues and act as individualized* in situ* vaccines to promote T-cell priming 109. Second, OVs stimulate the production of CXCL9 and CXCL10 and upregulate the expression of selectins and integrins, providing key signals for T-cell trafficking. In addition, the degradation of the ECM by OVs disrupts the physical barrier to T-cell infiltration 109. OVs can also deplete the immunosuppressive effects of CAFs, TAMs and MDSCs, significantly altering the TME 109.

Talimogene laherparepvec (T-VEC) was first shown to be effective as an oncolytic virotherapy for melanoma 110. Combination therapy with T-VEC and pembrolizumab increased CD8^+^ T-lymphocyte infiltration, IFN-γ expression and the therapeutic effect of PD-1 blockade in patients with advanced malignant melanoma 111. Similar effects have been observed with combination therapy of coxsackievirus and pembrolizumab 112. These studies suggest that combination therapy with OVs and ICIs can improve CD8^+^ T-cell infiltration and activation and help to overcome the resistance of cancer to ICIs in patients. Combined with T-cell therapy, promoting T-cell proliferation and infiltration into the local TME is a potential direction for the development of OVs.

### Chemotherapy and radiotherapy

It was previously thought that chemotherapy and radiotherapy exert their antitumor effects by directly killing tumor cells. However, accumulated evidence suggests that tumor suppression by chemotherapy and radiotherapy also relies on stimulating the immune system. When radiotherapy is administered to a local tumor, distant tumors outside the irradiated field also shrink. This phenomenon is termed the “abscopal effect”, which indicates the significance of the immune system in radiotherapy-mediated antitumor responses 113. After radiation therapy causes damage to tumor cells, ROS and endoplasmic reticulum (ER) stress mediate cellular stress and lead to ICDs 114. This cascade promotes DC activation, increases the production of TNFα and IL-1 and produces endogenous cancer vaccines *in vivo* 114. After radiation therapy, endothelial cells express ICAM1, VCAM1, and E-selectin, which facilitate the attraction of immune cells 114. Radiation also promotes the trafficking of effector T lymphocytes to the tumor site by inducing tumor cells to express and release chemokines (e.g., CXCL10 and CXCL16) 115. However, considering the adverse effects of radiation therapy, it is necessary to optimize the radiation dose and fractionation levels during radiation therapy. Fractionated radiotherapy at individual doses of less than 8-10 Gy helps to induce sufficient ICD without increasing hypoxia or immunosuppression, inducing a *de novo* antitumor response 115. In preclinical studies, stereotactic body radiotherapy (SBRT) increased effector T-cell infiltration in tumors and DLNs and was associated with higher survival rates 116.

In addition to radiotherapy, many chemotherapeutic agents can exert their immunostimulatory effects by enhancing immunogenicity and increasing T-cell infiltration 117. ICD inducing chemotherapy has been shown in various mouse models to contribute to the transformation of “cold tumors” to “hot tumors” in response to ICIs 118-120. New immunotherapies that reduce the toxic side effects of systemic chemotherapy and enhance the immunogenicity induced by chemotherapeutic agents deserve further exploration. A new cocktail therapy involves local chemoimmunotherapy by mixing chemotherapeutic agents with immune adjuvants and alginate (ALG). The ICD-inducing chemotherapeutic agents produced tumor vaccines *in situ* in response to adjuvant stimulation. *In situ* gelation of the drug adjuvant ALG enables slow release of the drug, thereby reducing systemic toxicity. The combination of ICIs further amplifies the immune response and inhibits tumor metastasis and recurrence 121.

### Local thermal ablation therapy

Image-guided thermal ablation has been developed as a promising method for the treatment of solid tumors. Currently, the commonly used thermal ablation methods include radiofrequency ablation (RFA), laser ablation (LA), microwave ablation (MWA), and high-intensity focused ultrasound (HIFU) ablation. RFA is widely used in the treatment of solid tumors, especially hepatocellular carcinoma (HCC). RFA utilizes the conversion of radiofrequency alternating current into heat to ablate the tissue surrounding the needle electrode and stimulate tumor-specific T-cell responses 122. However, problems with incomplete ablation and tumor recurrence are drawbacks of RFA. The combination of the tyrosine kinase inhibitor sunitinib and RFA improves HCC treatment. RFA caused the release of TSA *in situ* in tumors but caused the upregulation of PD-1 expression in T lymphocytes, which was related to the exhaustion of CD8^+^ T lymphocytes. Sunitinib inhibition of hepatocyte growth factor (HGF) inhibited the upregulation of PD-1 expression in tumor T lymphocytes. Inhibition of VEGF by sunitinib also promoted DC activation and inhibited tumor angiogenesis. Combination therapy ultimately led to a remarkable increase in CD8^+^ T lymphocytes and DCs as well as a decrease in Tregs, thus overcoming the drawbacks of monotherapy 123. In addition, HIFU, as a new minimally invasive ablation therapy, has become a hotspot of cancer treatment. HIFU delivers acoustic energy to the target tissues in a noninvasive and precise manner, generating high temperatures that cause coagulative necrosis of the tumor tissue. HIFU promotes ICD and the activation of T cells 124. HIFU also promotes antigen transfer to lymph nodes and T-cell migration to tumors through mechanical destruction of the mesenchyme 124.

## Therapeutic approaches to increase the number of antigen-specific T cells

### Adoptive cellular therapy (ACT)

ACT enhances the immune response of effector T cells to cancer, including tumor-infiltrating lymphocytes (TILs) and CAR-T cells 6. In TIL therapy, tumor-infiltrating lymphocytes are isolated from cancer patients, expanded *in vitro*, and then reinfused into the patient. However, due to the small number of infiltrating lymphocytes or the downregulation of MHC molecules, TILs are only used for the treatment of a few tumor types, such as malignant melanoma 125. CAR-T cells involve genetic modification of T lymphocytes to express the CAR to target tumor cells expressing a specific antigen 6. For example, CD19-specific CAR-T cells have become the gold standard for the treatment of B-cell malignancies. In addition, CAR-T cell therapy for leukemia and lymphoma was approved by the FDA in 2017 6. In contrast to TILs, CAR-T cells are not limited by the MHC and can further enhance the immune response to tumors through the addition of costimulatory domains (e.g., CD28, OX40, and 4-1BB) 6. This strategy leverages the direct recognition of tumor antigens by CAR-T cells and has the potential to treat “cold tumors” lacking pre-existing T-cell infiltration. CAR-T cells expressing IL-7 and CCL19 increased DC and T-cell infiltration in mouse solid tumor tissues and showed potent antitumor effects 126. In addition, the use of FLT3L-secreting CAR-T cells and immune adjuvants led to similar results and induced host T-cell antigen epitope spread 34. However, CAR-T cells exhibit limited clinical efficacy in solid tumors due to tumor antigen heterogeneity and insufficient infiltration into tumors 6. Additionally, the immunosuppressive microenvironment limits the tumor-killing effect of CAR-T cells, which makes it necessary to combine CAR-T cells with ICI therapy.

### Cancer vaccines

Therapeutic vaccines such as peptide and tumor cell vaccines, nucleotide vaccines encoding new epitopes, and dendritic cell vaccines have been encouraging clinical advances. Therapeutic vaccines expand the pool of tumor-specific T cells, increase the transport of T lymphocytes to tumor areas, and have become emerging modalities for immunotherapy 127. Sipuleucel-T, the first therapeutic cancer vaccine licensed by the FDA, has been used in the treatment of castration-resistant prostate cancer. Sipuleucel-T consists of the fusion protein PA2024 constructed from both prostatic acid phosphatase (PAP) and GM-CSF and autologous DCs, which enhance the antitumor effect 128. Tumor neoantigens are highly tumor-specific and immunogenic. The combination of the personalized neoantigen vaccine NEO-PV-01 with nivolumab significantly prolonged progression-free survival in an Ib clinical trial for patients with advanced malignant melanoma, NSCLC and bladder cancer. A neoantigen-specific T-cell response, T-cell trafficking to tumors and induction of epitope spread were also observed 129. The combination of the mRNA personalized cancer vaccine RO7198457 and atezolizumab showed clinical benefit in a phase Ib clinical trial for patients with advanced solid malignancies (NCT03289962). The vaccine induced a neoantigen-specific T-cell response in 77% of patients. This outcome demonstrates that the use of a personalized cancer vaccine combined with an immune checkpoint blockade can generate a specific immune response in patients. However, the complex operation, cumbersome process and high price remain limiting factors for the widespread use of personalized cancer vaccines in cancer treatment.

## Therapeutic approaches to promote T-cell trafficking and infiltration

### Oncogenic pathway inhibitors

The use of oncogenic pathway inhibitors helps reverse the inherent T-cell exclusion from tumors. PAK4 is abundantly expressed in “cold tumors” and plays a key role in the WNT/β-catenin pathway. Knocking down PAK4 or applying the PAK4 inhibitor KPT-9274 in a mouse tumor model enhanced CTL infiltration in tumors and improved the therapeutic efficacy of a PD-1 blockade 130. However, the efficacy of treatments targeting WNT remains controversial. For example, endogenous inhibitors of the WNT pathway, such as some proteins of the dickkopf (DKK) family, have a role in promoting tumor immune escape and are associated with a poorer prognosis in some cancers 131, 132. DKK2 inhibits WNT-β-catenin signaling by binding to the cell surface receptors LRP5 and LRP6 of the WNT pathway 133. DKK2 expression is upregulated in human colorectal cancers (CRCs) and promotes tumor progression by inhibiting the activation of NK cells and CD8^+^ T cells 131. This challenges the notion that inhibition of the WNT pathway will improve immunotherapy. Furthermore, recent studies have shown that activation of the WNT pathway in endothelial cells promotes T-cell infiltration into tumors and enhances the effectiveness of immunotherapies such as the ACT, suggesting that there is still a need to further investigate the feasibility of using WNT inhibitors as immune adjuvants 134. Indeed, clinical data using inhibitors of the WNT/βcatenin pathway do not support completely its putative function to boost immunotherapy in the clinic.

Activation of the PI3K-AKT pathway is associated with the inhibition of CTL infiltration and function. PI3Kβ inhibitors inhibit the activation of the AKT pathway in PTEN-deficient melanoma cell lines and enhance T-cell-mediated killing 38. The combination of PI3Kβ inhibitors and ICIs significantly increased the number of infiltrating T lymphocytes in murine tumor models 38.

RAS was considered as an “undruggable” target in the past 135. Monotherapies targeting the RAS oncogene have faced limited efficacy due to multiple mechanisms, such as feedback reactivation of the RAS downstream pathway 39. However, recent studies have found that ARS-1620, a small-molecule inhibitor that specifically targets KRAS-G12C mutants, significantly inhibited the growth of KRAS-G12C tumors 136. This evidence suggests a new therapeutic avenue to inhibiting mutant RAS. In addition, MEK inhibitors in combination with ICIs led to an increase in tumor-infiltrating T cells and a decrease in the percentage of MDSCs, which in turn significantly inhibited tumor growth in TP53/KRAS-driven lung cancer mouse models 137. Oncogenic mutations of BRAF activate the RAF-MEK-ERK (MAPK) pathway. Inhibition of BRAF or MEK inhibits the production of inhibitory cytokines (e.g., IL-6, IL-10, and VEGF) or enhances the expression of melanocyte differentiation antigen, thereby promoting melanoma recognition by T cells 138, 139. Three variant kinase inhibitors targeting MEK, cobimetinib, trametinib, and binimetinib, are clinically approved for therapeutic use in BRAF V600 mutant melanoma 135.

CDk4/6 can bind to cyclin D, which enables cells to enter S-phase through the RB-EF2 pathway and promotes tumor cell proliferation. Treatment of CT26 syngeneic mouse tumors with the CDK4/6 inhibitor abemaciclib led to an increase in tumor-infiltrating T lymphocytes, and a significant upregulation of T-cell activity, as evidenced by the increased expression of T-lymphocyte activation markers (e.g., IFNG, GZMB, CCL4 and CCL5). Abemaciclib also led to enhanced antigen presentation and had a synergistic effect when applied with anti-PD-1 therapy 44.

### Epigenetic modification inhibitors

Epigenetic therapies can transform tumors from the immune “cold” state to the immune “hot” state through a variety of mechanisms. Epigenetic drugs can enhance the expression of multiple chemokines, such as CXCL9, CXCXL10, and CCL5, and promote T-cell trafficking to tumors 48, 49, 51. Epigenetic therapy can also induce ERVs and suppress MYC signaling, thereby enhancing the expression of type I IFNs and related chemokines 51. In addition, epigenetic therapies can enhance tumor immunogenicity by increasing the expression of tumor antigens such as CTA and by restoring MHC-I antigen processing and presentation mechanisms 140, 141. The DNMT inhibitor guadecitabine upregulated MHC-I expression in breast tumor cells, enhanced IFN-γ responses, and promoted T-cell recruitment to tumors. In addition, guadecitabine had a synergistic effect with an anti-PD-L1 antibody 142. This outcome suggests the feasibility of combining epigenetic inhibitors with ICI strategies for future clinical application. A variety of epigenetic drugs have been approved by the FDA, such as azacitidine and decitabine (DNMT inhibitors), tazemetostat (an EZH2 inhibitor), and entinostat and vorinostat (HDAC inhibitors) 143.

### Antiangiogenic therapy

Persistent angiogenesis caused by a dysregulated balance between pro-and antiangiogenic signals is one of the hallmarks of tumors 144. Antiangiogenic therapy (AT) structurally and functionally overcomes tumor vascular abnormalities, improves tissue perfusion, and increases the infiltration of immune effector cells. AT-mediated immune reprogramming in turn improves vascular normalization, thereby creating an enhanced positive feedback loop 56, 145. Bevacizumab is the first FDA-approved angiogenesis inhibitor. Increased infiltration of tumor-specific T lymphocytes was observed after combination therapy with bevacizumab and atezolizumab in patients with metastatic renal cancer. In addition, combination therapy resulted in downregulation of the expression of vascular signature genes (e.g., ANGPT2 and CD31) and upregulation of CD8^+^ T effector genes (e.g., CD8A, GZMB, and IFNG) and MHC-I, as well as chemokines (e.g., fractalkine) 146. These results imply that the increase in tumor-specific T-cell infiltration may be due to enhanced lymphocyte trafficking mediated by the combination therapy. In addition, in the phase III IMbrave150 trial for patients with unresectable hepatocellular carcinoma, treatment with atezolizumab in combination with Bevacizumab significantly improved overall survival and progression-free survival outcomes compared with the results of sorafenib treatment 147. Given the relationship between vascular normalization and immune reprogramming, combination therapy is expected to further reverse the immunosuppressive microenvironment and improve the efficacy of immunotherapy.

### TGFβ inhibitors

Considering the immunosuppressive function of TGFβ, therapy based on the inhibition of TGFβ has been validated as an effective approach to promote T-lymphocyte infiltration. TGFβ is related to a lack of immune response in the noninflamed T-cell phenotype. In a mammary cancer mouse model with the immune-excluded phenotype, combined treatment with anti-PD-L1 and anti-TGFβ antibodies significantly reduced tumor burden and increased tumor-infiltrating T cells, especially CD8^+^ T effector cells 73. Galunisertib, a small molecule that inhibits the activity of TGFBR1 kinase, has been the most widely tested compound. Galunisertib treatment increased T-cell infiltration and improved susceptibility to checkpoint therapy in a mouse colorectal model 72. TGFβ impedes the generation of *in situ* tumor vaccines after radiotherapy. Treatment with the 1D11 antibody, which blocks systemic TGFβ activity, enhanced the initiation of T-cell responses to endogenous tumor antigens after subcutaneous tumor irradiation 148.

### CXCR4 inhibitors

CXCR4 is a receptor for CXCL12, which is overexpressed in a wide range of tumors. The CXCL12-CXCR4 axis plays an indirect role in the sequestration of CTLs from the tumor area to reduce CTL infiltration and mediates the infiltration of immunosuppressive cells into tumors 52. In the PDAC model, inhibition of CAF-mediated CXCL12/CXCR4 axis with the CXCR4 inhibitor AMD3100 promoted T-cell accumulation and cancer regression 84. Previous studies have shown that immunotherapies such as pembrolizumab were not effective against “cold tumors” such as pancreatic cancer. However, in the COMBAT trial, the synergistic treatment of metastatic PDAC with the CXCR4 antagonist BL8040 and pembrolizumab increased tumor-infiltrating CD8^+^ effector T lymphocytes, reduced the density of MDSCs in tumors, and reduced the number of circulating Tregs 149. These results reveal that regulating certain chemokines facilitates the homing of tumor-specific T lymphocytes to the tumor and reverses immune resistance.

## New cancer therapies based on nanotechnology

### Nanomedicine

**Figure 5:**
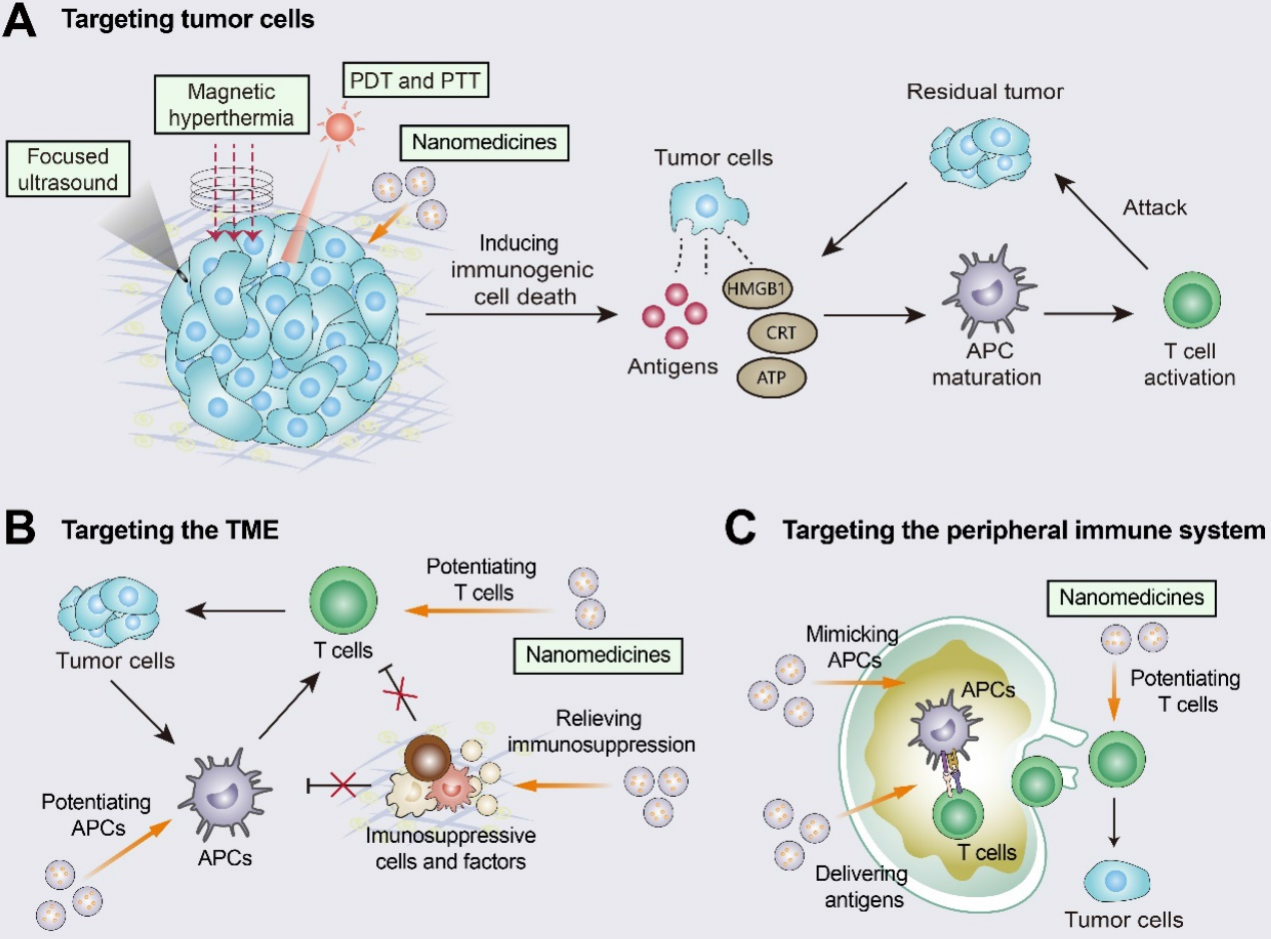
** Improving T-cell infiltration with nanomedicines.** Nanomedicines have three different targeting pathways: tumor cells, the TME, and the peripheral immune system. **(A)** Multiple approaches including photothermal therapy (PTT), photodynamic therapy (PDT), magnetic hyperthermia (MH), and high-intensity focused ultrasound (HIFU) can induce ICD by promoting the release of tumor antigens and damage-associated molecular patterns (DAMPs). The released DAMPs act as adjuvants to enhance the immunogenicity of the tumor and, together with the released tumor antigens, promote dendritic cell (DC) activation and T-cell priming. **(B)** When targeting the TME, nanomedicines inhibit immunosuppressive cells and immunosuppressive molecules (e.g., TGFβ) and enhance the activity of T cells. **(C)** When the peripheral immune system is targeted, nanomedicines are engineered to augment tumor antigen presentation and T-cell priming in lymph nodes. Adapted with permission from 150, copyright 2019 American Chemical Society.

A variety of nanomedicines combined with immunotherapy contribute to the transition from “cold tumors” to “hot tumors” (Figure 5). Nanomedicines have three different targeting pathways: tumor cells, the TME, and the peripheral immune system 150. Nanomedicine-based targeted tumor therapy includes passive and active targeting. Passive targeting can promote selective accumulation of nanomedicines in tumors through EPR effects 151. However, recent studies have suggested that the passive targeting ability of nanomaterials may be associated with transcytosis 152. Active targeting involves the use of targeted ligands (e.g., peptides, antibodies and transferrin) that specifically recognize specific receptors expressed by tumor cells 15.

Nanomedicines targeting tumor cells can induce ICD and enhance the tumor-immunity cycle 150, 153. For example, doxil, a PEGylated liposome of the chemotherapeutic drug doxorubicin, promoted DC and CD8^+^ T cell proliferation via ICD and inhibited Treg infiltration 154. Doxil was also synergistic with ICIs (anti-PD-1 and anti-CTLA-4) and showed higher efficacy than free doxorubicin 154. When targeting the TME, nanomedicines inhibit immunosuppressive cells (e.g., M2 TAMs and MDSCs) and immunosuppressive molecules (e.g., TGFβ and ADO), and they can also augment the activity and function of effector immune cells (e.g., macrophages and CTLs) 150, 155. The TGFβ receptor inhibitor SB525334 was loaded onto liposomes targeting ACT T cells. This nanomedicine inhibited TGFβ expression and promoted T-cell activation as well as tumor regression in melanoma mice 156. Loading liposomes with IL-2 and agonistic anti-4-1BB enhanced the tumor infiltration of CTLs as well as cytokine production and granzyme expression 157. When targeting the peripheral immune system, nanomedicines are designed to promote antigen presentation and CTL production in DLNs. Nanomedicine-based vaccines improved antigen delivery to lymph nodes, promoted antigen cross-presentation, and increased CTL activation levels 150, 158, 159. Furthermore, nanomedicines have been engineered to directly promote T-cell priming by replacing APCs in secondary lymph nodes. The use of biomimetic magnetosomes as artificial APCs was characterized by magnetic nanoclusters encapsulated by leukocyte membranes and modified to stimulate signals on the membranes. Artificial APCs not only expanded and stimulated CTLs, but also guided reinfused CTLs efficiently into tumor tissues, thus inhibiting tumor growth 160.

### Tumor phototherapy (PT)

PT, including photothermal therapy (PTT) and photodynamic therapy (PDT), has been developed as a potential treatment for solid tumors, especially superficial tumors. PTT uses photothermal agents (e.g., organic nanoparticles, gold nanoparticles and graphene oxide) with photothermal conversion capabilities to absorb near-infrared (NIR) lasers and convert them into heat energy to kill tumor cells 15. PDT involves the use of a laser to irradiate the tumor into which photosensitizers had been delivered to activate the photosensitizers and produce cytotoxic ROS. It can cause DNA damage in the nucleus and thus induce tumor cell death 15. Compared to other ablation modalities (e.g., radiofrequency ablation and microwave ablation), PT is more selective and less toxic to surrounding tissues due to the accumulation of photosensitizers in the tumor and the controllability of the light.

PTT and PDT induce ICD through thermal and chemical damage, respectively, and enhance the infiltration of CTLs and the immunotherapeutic response 15. PDT based on a layer-by-layer Apt/ PDGsˆs@pMOF nanoplatform enhanced the immunogenicity of triple-negative breast cancer in mice, selectively suppressed MDSCs, and promoted the transition to “hot tumors” 161. However, a single PT treatment shows limited efficacy because of limited light penetration, a heat-shock response due to the PTT, and the dependence of PDT on oxygen. The combination of PDT and PTT can achieve promising synergistic antitumor effects. A hybrid nanoporphyrin (Pp18-Lips)-mediated synergistic PTT/PDT caused an increase in tumor-infiltrating CTLs and inflammatory cytokines (e.g., TNF-α and IFN-γ) and a decrease in Tregs. Synergistic PDT/PTT produced a stronger antitumor immune response and stronger tumor suppression than PDT or PTT alone 162. PT is also synergistic with immunotherapy, as PT enhances the immunogenicity of the tumor by mediating ICD, while immunotherapy enhances the “abscopal effect” of the treatment. PTT-mediated GOP@aPD-1 nanoparticles efficiently delivered anti-PD-1 to melanoma cells and combined ICI treatment with tumor-targeted PTT. This combination resulted in increased CD8^+^ T-cell infiltration, elevated efficacy of the PD-1 blockade in mouse melanoma, and inhibition of tumor growth 163.

### Magnetic hyperthermia (MH)

MH refers to the selective heating of tumors by converting magnetic energy into thermal energy through the hysteresis and relaxation effects of magnetic nanoparticles (MNPs) in the presence of an alternating magnetic field (AMF) 164. In addition to killing tumor cells with heat, MH also induces ICD, showing great potential in cancer therapy. Compared to PT and thermal ablation therapy, MH has no penetration depth limit and enables more effective targeting and more precise control of the heating temperature. The iron oxide nanomedicine ferumoxytol promoted the polarization of macrophages into the proinflammatory M1 phenotype. This switch induced the apoptosis of tumor cells, as indicated by increased caspase-3 cleavage 165. Another typical example is ferrimagnetic vortex-domain iron oxide nanorings (FVIOs), which have excellent nanomagnetic properties. FVIO-mediated mild MH induced the expression of CRT on the surface of 4T1 breast tumor cells and promoted T-cell activation. FVIOs caused a significant increase in CTL infiltration along with a decrease in MDSCs and were synergistic with anti-PD-L1 antibodies 166.

### High-intensity focused ultrasound (HIFU)

The combination of HIFU and immunotherapy achieved significant therapeutic efficacy. Compared to immunotherapy alone or HIFU alone, the combination of HIFU and the TLR agonist CpG in mouse melanoma enhanced antigen cross-presentation in DLNs, release of type I IFNs, and expression of genes relevant to T-cell priming and stimulation (e.g., Eomes, Prf1 and Icos) 167. However, HIFU remains under-researched and there is a lack of studies demonstrating tumor control with HIFU monotherapy 124. The current state of the field suggests that HIFU can promote T-cell priming and tumor regression, but induction of additional immune adjuvants may be necessary. The killing of tumor cells by HIFU has also been linked to cavitation effects, which can efficiently aggregate energy. A recent study showed that direct injection of microbubbles and plasmid DNA encoding IFN-β into tumors in a mouse model followed by application of low-frequency ultrasound (250 kHz) to break up the tumors was shown to remove a substantial number of tumor cells and simultaneously achieve massive infiltration of CTLs. The remaining tumor cells also formed membrane pores, allowing gene transfection of the cells and triggering antitumor immune responses 168.

## Conclusions and future perspectives

Considering the relevance of T-lymphocyte infiltration in tumor sites to the prognosis of ICI therapy, the cause of the absence of a T-cell response is crucial to determine. In this review, we summarize various mechanisms that inhibit T-cell infiltration, such as defects in tumor antigen processing and presentation processes, endogenous oncogenic pathway activation, aberrant vasculature and chemokines, and TME suppression. Other known and unknown factors affecting T-cell infiltration, such as TLSs and the microbiome, remain to be evaluated. TLSs have a lymph node-like function and are relevant to T-cell infiltration into tumors and a good response to immunotherapy 169, 170. Exploring therapeutic strategies to enhance TLS formation and function may promote the activation of naïve T lymphocytes by DCs in close proximity to the tumor and improve the response to cancer immunotherapy. Compared to “cold tumors”, “hot tumors” are more responsive to ICI monotherapy. Thus, promoting the conversion of “cold tumors” to “hot tumors” through interventions can help reduce resistance to ICIs. In addition, we then discuss a variety of therapeutic measures to improve T-cell infiltration, such as oncogenic pathway inhibitors, anti-vascular therapy, ACT, vaccines, oncolytic viruses, and cytotoxic therapies. The combination of ICIs with these therapies reverses T-cell exhaustion, enhances the “abscopal effects” of therapy, and demonstrates incremental clinical efficacy. However, the optimal dose and sequence of administration of combination therapy needs to be further evaluated to optimize T-cell function, promote T-cell memory, and avoid overactivation 171. In addition, some issues still need to be addressed, such as the nonspecific distribution of drugs, and the treatment-induced systemic adverse effects.

**Figure 6:**
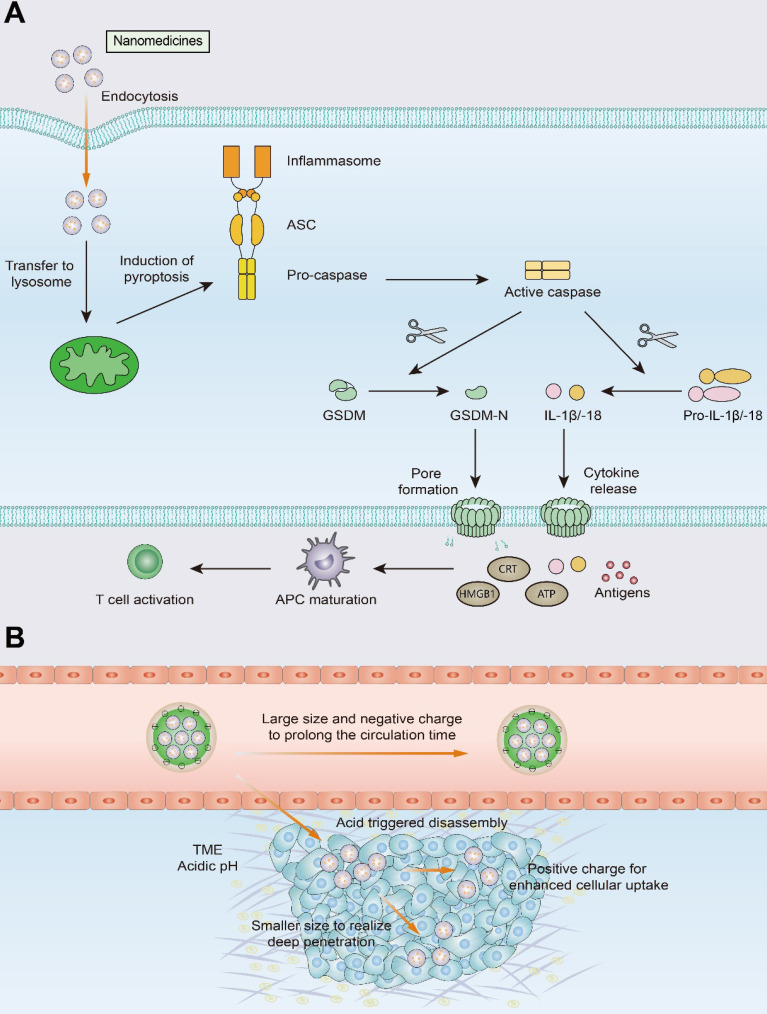
** Schematic illustration of pyroptosis and size-transformable nanoparticles. (A)** Multiple nanomedicines regulate the expression of caspase proteins that mediate the pyroptosis process. Activated caspases cut gasdermin (GSDM) into two fragments: the C-terminal domain and the N-terminal domain. Following cleavage, the gasdermin-N domains result in cell swelling with big bubbles. Gasdermin-induced pyroptosis results in the release of a massive quantity of proinflammatory molecules and activation of T cells. **(B)** Use of size-transformable nanoparticles to prolong the circulation time and realize deep penetration.

With the development of nanotechnology, the nanomedicine and biomaterial-assisted local therapies offers new opportunities for the future. The use of nanomedicines improves drug precision and bioavailability, reduces immunotherapy-induced side effects, and enables selective accumulation of drugs in tumors through EPR effects and active targeting. As mentioned above, PTT, PDT, MH, and HIFU are all capable of inducing a *de novo* antitumor response, which is achieved through the induction of ICD. These approaches do not require prior knowledge of tumor antigens and induce the generation of endogenous personalized *in situ* vaccines. However, killing solid tumors by noninflammatory apoptosis or ablation does not make tumor cells sufficiently immunogenic. In contrast to apoptosis, pyroptosis is a proinflammatory form of cell death that leads to the release of a massive quantity of inflammatory molecules (e.g., IL-1β and IL-18), mobilizing a robust antitumor T-cell response (Figure 6) 172-174. The use of nanotechnology to induce pyroptosis increases the immunogenicity of tumor cells and may effectively improve T-cell infiltration in tumors 172. For example, Zhao et al designed biomimetic nanoparticles (BNPs) loaded with indocyanine green (ICG) and decitabine for photoinduced pyroptosis. Due to promoter methylation of the GSDME gene, GSDME expression is much lower in most tumor cells than in normal cells. As an inhibitor of DNA methylation, decitabine promotes caspase-3 cleavage to GSDME by upregulating GSDME expression, thus leading to tumor cell pyroptosis. Pyroptosis mediated by BNP results in the release of a large number of inflammatory molecules from tumor cells and induces DC maturation and T-cell activation in DLNs, demonstrating a robust immune response against primary and distant tumors 175.

However, certain challenges to nanomedicine use must be overcome, such as short blood circulation time, and insufficient penetration and accumulation in tumor tissues. The use of size-transformable nanoparticles in phototherapy or chemotherapy may achieve deep penetration of nanomedicines and improve T-cell infiltration into tumors 176, 177. Nanocarriers with relatively large particle sizes utilize the EPR effect to prolong the circulation time of the nanomedicine and improve its accumulation in tumor tissue. Upon reaching the tumor site, the nanomedicine undergoes size transformation in response to pH or enzymes and releases transformed small nanoparticles that exhibit effective tumor tissue penetration and can be thus efficiently internalized by tumor cells.

In addition, the use of small molecular weight nanobodies in diagnostic imaging allows for a more convenient and complete assessment of the extent of T-cell infiltration in the TME, providing new ideas for achieving the integration of diagnosis and treatment of tumors. A better understanding of these aspects will be beneficial for guiding personalized cancer immunotherapy and extending the benefits of ICI therapy to a broader group of patients.

## Figures and Tables

**Table 1:** Mechanisms of defective T-cell priming

Mechanisms	Examples	References
Lack of tumor antigens	Lack of neoantigens	16
	Low mutational burden	16, 17
Insufficient antigen processing or presentation	Deletion of B2M	24, 25
	Deletion of TAP	23
	Loss of MHC class I (Lysosomal pathway)	26
Dysfunction of DC-T cell interaction	Loss of BATF3 DCs	32
	Impaired DC activation (Loss of FLT3L or GM-CSF)	35
	Overexpression of cosuppressive signals (CTLA-4)	28
	Overexpression of STC1	29

**Table 2:** Mechanisms of deficient T-cell homing to the tumor bed

Mechanisms	Examples	References
Oncogenic Pathway Activation	WNT/β-catenin activation	32, 36
	Loss of PTEN	38
	RAS activation	39, 138
	MYC activation	40
	LKB1 inactivation	42
Aberrant chemokine	Absence of CXCL9 and CXCL10	36
	Epigenetic regulation	48-50
	CXCL12 overexpression	52
Aberrant Vasculature	Downregulation of adhesion molecules (ICAM, VCAM, P-and E-selectin)	55-57
	VEGF overexpression	59
	FasL upregulation	60
	Pericyte abnormality	56, 61
Hypoxia	HIF-1	64-66
Immunosuppressive TME	TGFβ overexpression	72, 73
	ADO, IDO	68, 78
	CAF	82-84
Metabolic competition	High glycolytic activity	90-93
	Lactate accumulation and acidification	95, 96
	Cholesterol competition	98, 99

**Table 3:** Examples of therapeutic approaches to drive T cells into tumors

Main mechanisms	Therapeutic approaches	References
Promotes T-cell priming	Immune adjuvants (TLR agonists, STING agonists)	102, 105
	Oncolytic viruses	109, 111
	Chemotherapy and radiotherapy	114, 119
	Epigenetic modification inhibitors (DNMT inhibitor, HDAC inhibitor, EZH2 inhibitor)	140-142
	Metabolic intervention	94, 97, 99
	Local thermal ablation therapy (Radiofrequency ablation)	123
	Photothermal therapy and photodynamic therapy	161-163
	Magnetic hyperthermia	165, 166
	High-intensity focused ultrasound	167, 168
Promotes T-cell expansion	Adoptive cellular therapy (TILs, CAR-T cells)	34, 125, 126
	Vaccines	129
Promotes T-cell trafficking and infiltration	Oncogenic pathway inhibitors	38, 130, 138
	Epigenetic modification inhibitors	48, 49, 51
	Antiangiogenic therapy (anti-VEGF)	146
	TGFβ inhibitors	72, 73, 148
	CXCR4 inhibitors	84, 149

## Abbreviations

A2AR: A2A receptor
ADO: adenosine
ALG: alginate
AMF: alternating magnetic field
APC: antigen presenting cell
APM: antigen processing and presentation machinery
ASA: Acetylsalicylic acid
AT: Antiangiogenic therapy
ATP: adenosine triphosphate
B2M: beta-2-microglobulin
BNPs: biomimetic nanoparticles
CAFs: cancer-associated fibroblasts
CAR: chimeric antigen receptor
CDCs: conventional DCs
cGAS: cyclic GMP-AMP synthase
CLRs: C‑type lectin receptors
CRCs: colorectal cancers
CRT: calreticulin
CSF-1: colony-stimulating factor 1
CTAs: cancer/testis antigens
CTLA4: cytotoxic T lymphocyte-associated antigen-4
CTLs: cytotoxic T lymphocytes
CXCL: CXC-chemokine ligand
DAMPs: damage-associated molecular patterns
DCs: dendritic cells
DKK: dickkopf
DLNs: draining lymph nodes
DNMT: DNA methyltransferase
ECM: extracellular matrix
EPR: enhanced permeability and retention
ER: endoplasmic reticulum
ERV: endogenous retrovirus
ETBR: endothelin B receptor
EZH2: enhancer of zeste homolog 2
FLT3L: Fms-like tyrosine kinase 3 ligand
FVIOs: ferrimagnetic vortex-domain iron oxide nanorings
GLUT-1: glucose-transporter 1
GM-CSF: granulocyte-macrophage colony-stimulating factor
GSDM: gasdermin
HCC: hepatocellular carcinoma
HDAC: histone deacetylase
HEVs: high endothelial venules
HGF: hepatocyte growth factor
HIF1: Hypoxia-inducible factor 1
HIFU: high-intensity focused ultrasound
HMGB1: high mobility family protein B1
ICAMs: intercellular adhesion molecules
ICD: immunogenic cell death
ICG: indocyanine green
ICIs: immune checkpoint inhibitors
IDO: Indoleamine 2,3-dioxygenase
IFN-γ: interferon-γ
IL: interleukin
LA: laser ablation
MCT1: monocarboxylate transporter 1
MDSCs: myeloid-derived suppressor cells
MH: magnetic hyperthermia
MHC: major histocompatibility complex
MMP9: metalloproteinase-9
MNPs: magnetic nanoparticles
MWA: microwave ablation
NIR: near-infrared
NLRs: NOD‑like receptors
NSCLC: non-small cell lung cancer
OVs: oncolytic viruses
PAMPs: pathogen-associated molecular patterns
PAP: prostatic acid phosphatase
PD-1: programmed cell death protein 1
PD-L1: PD-1 ligand
PDAC: pancreatic ductal adenocarcinoma
PDCs: plasmacytoid DCs
PDT: photodynamic therapy
PGE2: prostaglandin E2
PRRs: pattern recognition receptors
PTT: photothermal therapy
RFA: radiofrequency ablation
RLRs: RIG‑I‑like receptors
SBRT: stereotactic body radiotherapy
SIRPα: signal-regulated protein-α
STC1: stanniocalcin 1
TAAs: tumor-associated antigens
TAMs: tumor-associated macrophages
TAP: transporter associated with antigen processing
TCGA: the Cancer Genome Atlas
TCR: T cell receptor
TGFβ: transforming growth factor-β
TILs: tumor-infiltrating lymphocytes
TLRs: Toll‑like receptors
TLSs: tertiary lymphoid structures
TMB: tumor mutational burden
TME: tumor microenvironment
TNF: tumor necrosis factor
Tregs: T-regulatory cells
TSAs: tumor-specific antigens
T-VEC: talimogene laherparepvec
VCAMs: vascular cell adhesion molecules
VEGF: vascular endothelial growth factor

## References

[1] Garon E, Rizvi N, Hui R, Leighl N, Balmanoukian A, Eder J (2015). Pembrolizumab for the treatment of non-small-cell lung cancer. N Engl J Med.

[2] Ferris R, Blumenschein G, Fayette J, Guigay J, Colevas A, Licitra L (2016). Nivolumab for Recurrent Squamous-Cell Carcinoma of the Head and Neck. N Engl J Med.

[3] Chen DS, Mellman I (2017). Elements of cancer immunity and the cancer-immune set point. Nature.

[4] Bruni D, Angell H, Galon J (2020). The immune contexture and Immunoscore in cancer prognosis and therapeutic efficacy. Nat Rev Cancer.

[5] Sharma P, Hu-Lieskovan S, Wargo JA, Ribas A (2017). Primary, Adaptive, and Acquired Resistance to Cancer Immunotherapy. Cell.

[6] June C, O'Connor R, Kawalekar O, Ghassemi S, Milone M (2018). CAR T cell immunotherapy for human cancer. Science.

[7] Hegde P, Karanikas V, Evers S (2016). The Where, the When, and the How of Immune Monitoring for Cancer Immunotherapies in the Era of Checkpoint Inhibition. Clin Cancer Res.

[8] Galon J, Bruni D (2019). Approaches to treat immune hot, altered and cold tumours with combination immunotherapies. Nat Rev Drug Discov.

[9] Herbst R, Soria J, Kowanetz M, Fine G, Hamid O, Gordon M (2014). Predictive correlates of response to the anti-PD-L1 antibody MPDL3280A in cancer patients. Nature.

[10] Hegde PS, Chen DS (2020). Top 10 Challenges in Cancer Immunotherapy. Immunity.

[11] Chen DS, Mellman I (2013). Oncology meets immunology: the cancer-immunity cycle. Immunity.

[12] Joyce J, Fearon D (2015). T cell exclusion, immune privilege, and the tumor microenvironment. Science.

[13] Dieckmann NM, Frazer GL, Asano Y, Stinchcombe JC, Griffiths GM (2016). The cytotoxic T lymphocyte immune synapse at a glance. J Cell Sci.

[14] Tang R, Xu J, Zhang B, Liu J, Liang C, Hua J (2020). Ferroptosis, necroptosis, and pyroptosis in anticancer immunity. J Hematol Oncol.

[15] Li X, Lovell JF, Yoon J, Chen X (2020). Clinical development and potential of photothermal and photodynamic therapies for cancer. Nat Rev Clin Oncol.

[16] Havel JJ, Chowell D, Chan TA (2019). The evolving landscape of biomarkers for checkpoint inhibitor immunotherapy. Nat Rev Cancer.

[17] Chan T, Yarchoan M, Jaffee E, Swanton C, Quezada S, Stenzinger A (2019). Development of tumor mutation burden as an immunotherapy biomarker: utility for the oncology clinic. Ann Oncol.

[18] Ready N, Hellmann M, Awad M, Otterson G, Gutierrez M, Gainor J (2019). First-Line Nivolumab Plus Ipilimumab in Advanced Non-Small-Cell Lung Cancer (CheckMate 568): Outcomes by Programmed Death Ligand 1 and Tumor Mutational Burden as Biomarkers. Journal of clinical oncology: official journal of the American Society of Clinical Oncology.

[19] Samstein R, Lee C, Shoushtari A, Hellmann M, Shen R, Janjigian Y (2019). Tumor mutational load predicts survival after immunotherapy across multiple cancer types. Nat Genet.

[20] Mouw K, Goldberg M, Konstantinopoulos P, D'Andrea A (2017). DNA Damage and Repair Biomarkers of Immunotherapy Response. Cancer Discov.

[21] McGrail DJ, Federico L, Li Y, Dai H, Lu Y, Mills GB (2018). Multi-omics analysis reveals neoantigen-independent immune cell infiltration in copy-number driven cancers. Nat Commun.

[22] Spranger S, Luke J, Bao R, Zha Y, Hernandez K, Li Y (2016). Density of immunogenic antigens does not explain the presence or absence of the T-cell-inflamed tumor microenvironment in melanoma. Proc Natl Acad Sci U S A.

[23] Seliger B (2008). Molecular mechanisms of MHC class I abnormalities and APM components in human tumors. Cancer Immunol Immunother.

[24] Torrejon D, Abril-Rodriguez G, Champhekar A, Tsoi J, Campbell K, Kalbasi A (2020). Overcoming Genetically Based Resistance Mechanisms to PD-1 Blockade. Cancer Discov.

[25] Gettinger S, Choi J, Hastings K, Truini A, Datar I, Sowell R (2017). Impaired HLA Class I Antigen Processing and Presentation as a Mechanism of Acquired Resistance to Immune Checkpoint Inhibitors in Lung Cancer. Cancer Discov.

[26] Yamamoto K, Venida A, Yano J, Biancur D, Kakiuchi M, Gupta S (2020). Autophagy promotes immune evasion of pancreatic cancer by degrading MHC-I. Nature.

[27] Takeuchi O, Akira S (2010). Pattern recognition receptors and inflammation. Cell.

[28] Logue E, Sha W (2004). CD28-B7 bidirectional signaling: a two-way street to activation. Nat Immunol.

[29] Lin H, Kryczek I, Li S, Green M, Ali A, Hamasha R (2021). Stanniocalcin 1 is a phagocytosis checkpoint driving tumor immune resistance. Cancer Cell.

[30] Haniffa M, Bigley V, Collin M (2015). Human mononuclear phagocyte system reunited. Semin Cell Dev Biol.

[31] Spranger S, Gajewski T (2018). Impact of oncogenic pathways on evasion of antitumour immune responses. Nat Rev Cancer.

[32] Spranger S, Dai D, Horton B, Gajewski TF (2017). Tumor-Residing Batf3 Dendritic Cells Are Required for Effector T Cell Trafficking and Adoptive T Cell Therapy. Cancer Cell.

[33] Demaria O, Cornen S, Daron M, Morel Y, Medzhitov R, Vivier E (2019). Harnessing innate immunity in cancer therapy. Nature.

[34] Lai J, Mardiana S, House I, Sek K, Henderson M, Giuffrida L (2020). Adoptive cellular therapy with T cells expressing the dendritic cell growth factor Flt3L drives epitope spreading and antitumor immunity. Nat Immunol.

[35] Kingston D, Schmid M, Onai N, Obata-Onai A, Baumjohann D, Manz M (2009). The concerted action of GM-CSF and Flt3-ligand on *in vivo* dendritic cell homeostasis. Blood.

[36] Spranger S, Bao R, Gajewski T (2015). Melanoma-intrinsic β-catenin signalling prevents anti-tumour immunity. Nature.

[37] Grasso C, Giannakis M, Wells D, Hamada T, Mu X, Quist M (2018). Genetic Mechanisms of Immune Evasion in Colorectal Cancer. Cancer Discov.

[38] Peng W, Chen J, Liu C, Malu S, Creasy C, Tetzlaff M (2016). Loss of PTEN Promotes Resistance to T Cell-Mediated Immunotherapy. Cancer Discov.

[39] Hamarsheh S, Groß O, Brummer T, Zeiser R (2020). Immune modulatory effects of oncogenic KRAS in cancer. Nat Commun.

[40] Jaiswal S, Jamieson C, Pang W, Park C, Chao M, Majeti R (2009). CD47 is upregulated on circulating hematopoietic stem cells and leukemia cells to avoid phagocytosis. Cell.

[41] Kortlever R, Sodir N, Wilson C, Burkhart D, Pellegrinet L, Brown Swigart L (2017). Myc Cooperates with Ras by Programming Inflammation and Immune Suppression. Cell.

[42] Koyama S, Akbay E, Li Y, Aref A, Skoulidis F, Herter-Sprie G (2016). STK11/LKB1 Deficiency Promotes Neutrophil Recruitment and Proinflammatory Cytokine Production to Suppress T-cell Activity in the Lung Tumor Microenvironment. Cancer Res.

[43] Jerby-Arnon L, Shah P, Cuoco M, Rodman C, Su M, Melms J (2018). A Cancer Cell Program Promotes T Cell Exclusion and Resistance to Checkpoint Blockade. Cell.

[44] Schaer D, Beckmann R, Dempsey J, Huber L, Forest A, Amaladas N (2018). The CDK4/6 Inhibitor Abemaciclib Induces a T Cell Inflamed Tumor Microenvironment and Enhances the Efficacy of PD-L1 Checkpoint Blockade. Cell Rep.

[45] Burdelya L, Kujawski M, Niu G, Zhong B, Wang T, Zhang S (2005). Stat3 activity in melanoma cells affects migration of immune effector cells and nitric oxide-mediated antitumor effects. J Immunol.

[46] van der Woude LL, Gorris MAJ, Halilovic A, Figdor CG, de Vries IJM (2017). Migrating into the Tumor: a Roadmap for T Cells. Trends Cancer.

[47] Maimela NR, Liu S, Zhang Y (2019). Fates of CD8+ T cells in Tumor Microenvironment. Comput Struct Biotechnol J.

[48] Peng D, Kryczek I, Nagarsheth N, Zhao L, Wei S, Wang W (2015). Epigenetic silencing of TH1-type chemokines shapes tumour immunity and immunotherapy. Nature.

[49] Nagarsheth N, Peng D, Kryczek I, Wu K, Li W, Zhao E (2016). PRC2 Epigenetically Silences Th1-Type Chemokines to Suppress Effector T-Cell Trafficking in Colon Cancer. Cancer Res.

[50] Dangaj D, Bruand M, Grimm A, Ronet C, Barras D, Duttagupta P (2019). Cooperation between Constitutive and Inducible Chemokines Enables T Cell Engraftment and Immune Attack in Solid Tumors. Cancer Cell.

[51] Topper M, Vaz M, Chiappinelli K, DeStefano Shields C, Niknafs N, Yen R (2017). Epigenetic Therapy Ties MYC Depletion to Reversing Immune Evasion and Treating Lung Cancer. Cell.

[52] Mortezaee K (2020). CXCL12/CXCR4 axis in the microenvironment of solid tumors: A critical mediator of metastasis. Life Sci.

[53] Yuen K, Liu L, Gupta V, Madireddi S, Keerthivasan S, Li C (2020). High systemic and tumor-associated IL-8 correlates with reduced clinical benefit of PD-L1 blockade. Nat Med.

[54] Schalper K, Carleton M, Zhou M, Chen T, Feng Y, Huang S (2020). Elevated serum interleukin-8 is associated with enhanced intratumor neutrophils and reduced clinical benefit of immune-checkpoint inhibitors. Nat Med.

[55] Georganaki M, van Hooren L, Dimberg A (2018). Vascular Targeting to Increase the Efficiency of Immune Checkpoint Blockade in Cancer. Front Immunol.

[56] Huang Y, Kim BYS, Chan CK, Hahn SM, Weissman IL, Jiang W (2018). Improving immune-vascular crosstalk for cancer immunotherapy. Nat Rev Immunol.

[57] Muller W (2011). Mechanisms of leukocyte transendothelial migration. Annu Rev Pathol.

[58] Buckanovich R, Facciabene A, Kim S, Benencia F, Sasaroli D, Balint K (2008). Endothelin B receptor mediates the endothelial barrier to T cell homing to tumors and disables immune therapy. Nat Med.

[59] Apte R, Chen D, Ferrara N (2019). VEGF in Signaling and Disease: Beyond Discovery and Development. Cell.

[60] Motz G, Santoro S, Wang L, Garrabrant T, Lastra R, Hagemann I (2014). Tumor endothelium FasL establishes a selective immune barrier promoting tolerance in tumors. Nat Med.

[61] Baluk P, Hashizume H, McDonald D (2005). Cellular abnormalities of blood vessels as targets in cancer. Curr Opin Genet Dev.

[62] Colbeck E, Ager A, Gallimore A, Jones G (2017). Tertiary Lymphoid Structures in Cancer: Drivers of Antitumor Immunity, Immunosuppression, or Bystander Sentinels in Disease?. Front Immunol.

[63] McDonald P, Chafe S, Dedhar S (2016). Overcoming Hypoxia-Mediated Tumor Progression: Combinatorial Approaches Targeting pH Regulation, Angiogenesis and Immune Dysfunction. Front Cell Dev Biol.

[64] Semenza G (2012). Hypoxia-inducible factors in physiology and medicine. Cell.

[65] Damgaci S, Ibrahim-Hashim A, Enriquez-Navas P, Pilon-Thomas S, Guvenis A, Gillies R (2018). Hypoxia and acidosis: immune suppressors and therapeutic targets. Immunology.

[66] Facciabene A, Peng X, Hagemann I, Balint K, Barchetti A, Wang L (2011). Tumour hypoxia promotes tolerance and angiogenesis via CCL28 and T(reg) cells. Nature.

[67] Allard B, Allard D, Buisseret L, Stagg J (2020). The adenosine pathway in immuno-oncology. Nat Rev Clin Oncol.

[68] Antonioli L, Blandizzi C, Pacher P, Haskó G (2013). Immunity, inflammation and cancer: a leading role for adenosine. Nat Rev Cancer.

[69] Sek K, Mølck C, Stewart G, Kats L, Darcy P, Beavis P (2018). Targeting Adenosine Receptor Signaling in Cancer Immunotherapy. Int J Mol Sci.

[70] Young A, Ngiow S, Madore J, Reinhardt J, Landsberg J, Chitsazan A (2017). Targeting Adenosine in BRAF-Mutant Melanoma Reduces Tumor Growth and Metastasis. Cancer Res.

[71] Vigano S, Alatzoglou D, Irving M, Ménétrier-Caux C, Caux C, Romero P (2019). Targeting Adenosine in Cancer Immunotherapy to Enhance T-Cell Function. Front Immunol.

[72] Tauriello D, Palomo-Ponce S, Stork D, Berenguer-Llergo A, Badia-Ramentol J, Iglesias M (2018). TGFβ drives immune evasion in genetically reconstituted colon cancer metastasis. Nature.

[73] Mariathasan S, Turley S, Nickles D, Castiglioni A, Yuen K, Wang Y (2018). TGFβ attenuates tumour response to PD-L1 blockade by contributing to exclusion of T cells. Nature.

[74] Brabletz T, Pfeuffer I, Schorr E, Siebelt F, Wirth T, Serfling E (1993). Transforming growth factor beta and cyclosporin A inhibit the inducible activity of the interleukin-2 gene in T cells through a noncanonical octamer-binding site. Mol Cell Biol.

[75] Josefowicz S, Lu L, Rudensky A (2012). Regulatory T cells: mechanisms of differentiation and function. Annu Rev Immunol.

[76] Shimabukuro-Vornhagen A, Draube A, Liebig T, Rothe A, Kochanek M, von Bergwelt-Baildon M (2012). The immunosuppressive factors IL-10, TGF-β, and VEGF do not affect the antigen-presenting function of CD40-activated B cells. J Exp Clin Cancer Res.

[77] Li X, Wenes M, Romero P, Huang SC, Fendt SM, Ho PC (2019). Navigating metabolic pathways to enhance antitumour immunity and immunotherapy. Nat Rev Clin Oncol.

[78] Holmgaard R, Zamarin D, Li Y, Gasmi B, Munn D, Allison J (2015). Tumor-Expressed IDO Recruits and Activates MDSCs in a Treg-Dependent Manner. Cell Rep.

[79] Prendergast GC, Mondal A, Dey S, Laury-Kleintop LD, Muller AJ (2018). Inflammatory Reprogramming with IDO1 Inhibitors: Turning Immunologically Unresponsive 'Cold' Tumors 'Hot'. Trends Cancer.

[80] Long G, Dummer R, Hamid O, Gajewski T, Caglevic C, Dalle S (2019). Epacadostat plus pembrolizumab versus placebo plus pembrolizumab in patients with unresectable or metastatic melanoma (ECHO-301/KEYNOTE-252): a phase 3, randomised, double-blind study. Lancet Oncol.

[81] Szot C, Saha S, Zhang X, Zhu Z, Hilton M, Morris K (2018). Tumor stroma-targeted antibody-drug conjugate triggers localized anticancer drug release. J Clin Invest.

[82] Sahai E, Astsaturov I, Cukierman E, DeNardo DG, Egeblad M, Evans RM (2020). A framework for advancing our understanding of cancer-associated fibroblasts. Nat Rev Cancer.

[83] Salmon H, Franciszkiewicz K, Damotte D, Dieu-Nosjean M, Validire P, Trautmann A (2012). Matrix architecture defines the preferential localization and migration of T cells into the stroma of human lung tumors. J Clin Invest.

[84] Feig C, Jones J, Kraman M, Wells R, Deonarine A, Chan D (2013). Targeting CXCL12 from FAP-expressing carcinoma-associated fibroblasts synergizes with anti-PD-L1 immunotherapy in pancreatic cancer. Proc Natl Acad Sci U S A.

[85] Stylianopoulos T, Munn L, Jain R (2018). Reengineering the Physical Microenvironment of Tumors to Improve Drug Delivery and Efficacy: From Mathematical Modeling to Bench to Bedside. Trends Cancer.

[86] Mantovani A, Marchesi F, Malesci A, Laghi L, Allavena P (2017). Tumour-associated macrophages as treatment targets in oncology. Nat Rev Clin Oncol.

[87] DeNardo D, Ruffell B (2019). Macrophages as regulators of tumour immunity and immunotherapy. Nat Rev Immunol.

[88] Lin E, Pollard J (2007). Tumor-associated macrophages press the angiogenic switch in breast cancer. Cancer Res.

[89] Xia Y, Rao L, Yao H, Wang Z, Ning P, Chen X (2020). Engineering Macrophages for Cancer Immunotherapy and Drug Delivery. Adv Mater.

[90] Chang C, Qiu J, O'Sullivan D, Buck M, Noguchi T, Curtis J (2015). Metabolic Competition in the Tumor Microenvironment Is a Driver of Cancer Progression. Cell.

[91] Siska P, Singer K, Evert K, Renner K, Kreutz M (2020). The immunological Warburg effect: Can a metabolic-tumor-stroma score (MeTS) guide cancer immunotherapy?. Immunol Rev.

[92] Singer K, Kastenberger M, Gottfried E, Hammerschmied C, Büttner M, Aigner M (2011). Warburg phenotype in renal cell carcinoma: high expression of glucose-transporter 1 (GLUT-1) correlates with low CD8(+) T-cell infiltration in the tumor. Int J Cancer.

[93] Ottensmeier C, Perry K, Harden E, Stasakova J, Jenei V, Fleming J (2016). Upregulated Glucose Metabolism Correlates Inversely with CD8+ T-cell Infiltration and Survival in Squamous Cell Carcinoma. Cancer Res.

[94] Seth P, Csizmadia E, Hedblom A, Vuerich M, Xie H, Li M (2017). Deletion of Lactate Dehydrogenase-A in Myeloid Cells Triggers Antitumor Immunity. Cancer Res.

[95] Gottfried E, Kunz-Schughart L, Ebner S, Mueller-Klieser W, Hoves S, Andreesen R (2006). Tumor-derived lactic acid modulates dendritic cell activation and antigen expression. Blood.

[96] Wang J, Choi S, Niu X, Kang N, Xue H, Killam J (2020). Lactic Acid and an Acidic Tumor Microenvironment suppress Anticancer Immunity. Int J Mol Sci.

[97] Pilon-Thomas S, Kodumudi K, El-Kenawi A, Russell S, Weber A, Luddy K (2016). Neutralization of Tumor Acidity Improves Antitumor Responses to Immunotherapy. Cancer Res.

[98] Yang W, Bai Y, Xiong Y, Zhang J, Chen S, Zheng X (2016). Potentiating the antitumour response of CD8(+) T cells by modulating cholesterol metabolism. Nature.

[99] Liu X, Bao X, Hu M, Chang H, Jiao M, Cheng J (2020). Inhibition of PCSK9 potentiates immune checkpoint therapy for cancer. Nature.

[100] Cheon H, Borden E, Stark G (2014). Interferons and their stimulated genes in the tumor microenvironment. Semin Oncol.

[101] Nuhn L, De Koker S, Van Lint S, Zhong Z, Catani J, Combes F (2018). Nanoparticle-Conjugate TLR7/8 Agonist Localized Immunotherapy Provokes Safe Antitumoral Responses. Adv Mater.

[102] Ribas A, Medina T, Kummar S, Amin A, Kalbasi A, Drabick J (2018). SD-101 in Combination with Pembrolizumab in Advanced Melanoma: Results of a Phase Ib, Multicenter Study. Cancer Discov.

[103] Barber GN (2015). STING: infection, inflammation and cancer. Nat Rev Immunol.

[104] Reisländer T, Groelly F, Tarsounas M (2020). DNA Damage and Cancer Immunotherapy: A STING in the Tale. Mol Cell.

[105] Chin E, Yu C, Vartabedian V, Jia Y, Kumar M, Gamo A (2020). Antitumor activity of a systemic STING-activating non-nucleotide cGAMP mimetic. Science.

[106] Pan B, Perera S, Piesvaux J, Presland J, Schroeder G, Cumming J (2020). An orally available non-nucleotide STING agonist with antitumor activity. Science.

[107] Russell L, Peng KW, Russell SJ, Diaz RM (2019). Oncolytic Viruses: Priming Time for Cancer Immunotherapy. Biodrugs.

[108] Krysko D, Garg A, Kaczmarek A, Krysko O, Agostinis P, Vandenabeele P (2012). Immunogenic cell death and DAMPs in cancer therapy. Nat Rev Cancer.

[109] Twumasi-Boateng K, Pettigrew JL, Kwok YYE, Bell JC, Nelson BH (2018). Oncolytic viruses as engineering platforms for combination immunotherapy. Nat Rev Cancer.

[110] Andtbacka R, Kaufman H, Collichio F, Amatruda T, Senzer N, Chesney J (2015). Talimogene Laherparepvec Improves Durable Response Rate in Patients With Advanced Melanoma. J Clin Oncol.

[111] Ribas A, Dummer R, Puzanov I, VanderWalde A, Andtbacka R, Michielin O (2017). Oncolytic Virotherapy Promotes Intratumoral T Cell Infiltration and Improves Anti-PD-1 Immunotherapy. Cell.

[112] Kaufman HL, Spencer K, Mehnert J, Silk A, Wang J, Zloza A (2016). Phase Ib study of intratumoral oncolytic coxsackievirus A21 (CVA21) and pembrolizumab in subjects with advanced melanoma. Ann Oncol.

[113] Ngwa W, Irabor O, Schoenfeld J, Hesser J, Demaria S, Formenti S (2018). Using immunotherapy to boost the abscopal effect. Nat Rev Cancer.

[114] Barker H, Paget J, Khan A, Harrington K (2015). The tumour microenvironment after radiotherapy: mechanisms of resistance and recurrence. Nat Rev Cancer.

[115] Demaria S, Golden E, Formenti S (2015). Role of Local Radiation Therapy in Cancer Immunotherapy. JAMA oncology.

[116] Brooks E, Schoenhals J, Tang C, Micevic G, Gomez D, Chang J (2016). Stereotactic Ablative Radiation Therapy Combined With Immunotherapy for Solid Tumors. Cancer J.

[117] Galluzzi L, Humeau J, Buqué A, Zitvogel L, Kroemer G (2020). Immunostimulation with chemotherapy in the era of immune checkpoint inhibitors. Nat Rev Clin Oncol.

[118] Liu P, Zhao L, Pol J, Levesque S, Petrazzuolo A, Pfirschke C (2019). Crizotinib-induced immunogenic cell death in non-small cell lung cancer. Nat Commun.

[119] Yamazaki T, Buqué A, Ames T, Galluzzi L (2020). PT-112 induces immunogenic cell death and synergizes with immune checkpoint blockers in mouse tumor models. Oncoimmunology.

[120] Wang C, Wang J, Zhang X, Yu S, Wen D, Hu Q (2018). *In situ* formed reactive oxygen species-responsive scaffold with gemcitabine and checkpoint inhibitor for combination therapy. Sci Transl Med.

[121] Chao Y, Liang C, Tao H, Du Y, Wu D, Dong Z (2020). Localized cocktail chemoimmunotherapy after *in situ* gelation to trigger robust systemic antitumor immune responses. Sci Adv.

[122] Mizukoshi E, Yamashita T, Arai K, Sunagozaka H, Ueda T, Arihara F (2013). Enhancement of tumor-associated antigen-specific T cell responses by radiofrequency ablation of hepatocellular carcinoma. Hepatology.

[123] Qi X, Yang M, Ma L, Sauer M, Avella D, Kaifi J (2020). Synergizing sunitinib and radiofrequency ablation to treat hepatocellular cancer by triggering the antitumor immune response. J Immunother Cancer.

[124] Sheybani ND, Price RJ (2019). Perspectives on Recent Progress in Focused Ultrasound Immunotherapy. Theranostics.

[125] Rosenberg S, Yang J, Sherry R, Kammula U, Hughes M, Phan G (2011). Durable complete responses in heavily pretreated patients with metastatic melanoma using T-cell transfer immunotherapy. Clin Cancer Res.

[126] Adachi K, Kano Y, Nagai T, Okuyama N, Sakoda Y, Tamada K (2018). IL-7 and CCL19 expression in CAR-T cells improves immune cell infiltration and CAR-T cell survival in the tumor. Nat Biotechnol.

[127] van der Burg SH, Arens R, Ossendorp F, van Hall T, Melief CJM (2016). Vaccines for established cancer: overcoming the challenges posed by immune evasion. Nat Rev Cancer.

[128] Kantoff PW, Higano CS, Shore ND, Berger ER, Small EJ, Penson DF (2010). Sipuleucel-T Immunotherapy for Castration-Resistant Prostate Cancer. N Engl J Med.

[129] Ott P, Hu-Lieskovan S, Chmielowski B, Govindan R, Naing A, Bhardwaj N (2020). A Phase Ib Trial of Personalized Neoantigen Therapy Plus Anti-PD-1 in Patients with Advanced Melanoma, Non-small Cell Lung Cancer, or Bladder Cancer. Cell.

[130] Abril-Rodriguez G, Torrejon DY, Liu W, Zaretsky JM, Nowicki TS, Tsoi J (2020). PAK4 inhibition improves PD-1 blockade immunotherapy. Nat Cancer.

[131] Xiao Q, Wu J, Wang W, Chen S, Zheng Y, Yu X (2018). DKK2 imparts tumor immunity evasion through β-catenin-independent suppression of cytotoxic immune-cell activation. Nat Med.

[132] Malladi S, Macalinao D, Jin X, He L, Basnet H, Zou Y (2016). Metastatic Latency and Immune Evasion through Autocrine Inhibition of WNT. Cell.

[133] Bugter J, Fenderico N, Maurice M (2021). Mutations and mechanisms of WNT pathway tumour suppressors in cancer. Nat Rev Cancer.

[134] Carmona-Rodríguez L, Martínez-Rey D, Fernández-Aceñero M, González-Martín A, Paz-Cabezas M, Rodríguez-Rodríguez N (2020). SOD3 induces a HIF-2α-dependent program in endothelial cells that provides a selective signal for tumor infiltration by T cells. J Immunother Cancer.

[135] Moore A, Rosenberg S, McCormick F, Malek S (2020). RAS-targeted therapies: is the undruggable drugged?. Nat Rev Drug Discov.

[136] Janes M, Zhang J, Li L, Hansen R, Peters U, Guo X (2018). Targeting KRAS Mutant Cancers with a Covalent G12C-Specific Inhibitor. Cell.

[137] Lee J, Zhang Y, Eoh K, Sharma R, Sanmamed M, Wu J (2019). The Combination of MEK Inhibitor With Immunomodulatory Antibodies Targeting Programmed Death 1 and Programmed Death Ligand 1 Results in Prolonged Survival in Kras/p53-Driven Lung Cancer. J Thorac Oncol.

[138] Sumimoto H, Imabayashi F, Iwata T, Kawakami Y (2006). The BRAF-MAPK signaling pathway is essential for cancer-immune evasion in human melanoma cells. J Exp Med.

[139] Boni A, Cogdill A, Dang P, Udayakumar D, Njauw C, Sloss C (2010). Selective BRAFV600E inhibition enhances T-cell recognition of melanoma without affecting lymphocyte function. Cancer Res.

[140] Maatouk D, Kellam L, Mann M, Lei H, Li E, Bartolomei M (2006). DNA methylation is a primary mechanism for silencing postmigratory primordial germ cell genes in both germ cell and somatic cell lineages. Development.

[141] Ritter C, Fan K, Paschen A, Reker Hardrup S, Ferrone S, Nghiem P (2017). Epigenetic priming restores the HLA class-I antigen processing machinery expression in Merkel cell carcinoma. Sci Rep.

[142] Luo N, Nixon M, Gonzalez-Ericsson P, Sanchez V, Opalenik S, Li H (2018). DNA methyltransferase inhibition upregulates MHC-I to potentiate cytotoxic T lymphocyte responses in breast cancer. Nat Commun.

[143] Topper M, Vaz M, Marrone K, Brahmer J, Baylin S (2020). The emerging role of epigenetic therapeutics in immuno-oncology. Nat Rev Clin Oncol.

[144] Hanahan D, Weinberg R (2011). Hallmarks of cancer: the next generation. Cell.

[145] Liu Z, Wang Y, Huang Y, Kim B, Shan H, Wu D (2019). Tumor Vasculatures: A New Target for Cancer Immunotherapy. Trends Pharmacol Sci.

[146] Wallin J, Bendell J, Funke R, Sznol M, Korski K, Jones S (2016). Atezolizumab in combination with bevacizumab enhances antigen-specific T-cell migration in metastatic renal cell carcinoma. Nat Commun.

[147] Finn RS, Qin S, Ikeda M, Galle PR, Ducreux M, Kim T-Y (2020). Atezolizumab plus Bevacizumab in Unresectable Hepatocellular Carcinoma. N Engl J Med.

[148] Vanpouille-Box C, Diamond J, Pilones K, Zavadil J, Babb J, Formenti S (2015). TGFβ Is a Master Regulator of Radiation Therapy-Induced Antitumor Immunity. Cancer Res.

[149] Bockorny B, Semenisty V, Macarulla T, Borazanci E, Wolpin BM, Stemmer SM (2020). BL-8040, a CXCR4 antagonist, in combination with pembrolizumab and chemotherapy for pancreatic cancer: the COMBAT trial. Nat Med.

[150] Shi Y, Lammers T (2019). Combining Nanomedicine and Immunotherapy. Acc Chem Res.

[151] Irvine D, Dane E (2020). Enhancing cancer immunotherapy with nanomedicine. Nat Rev Immunol.

[152] Sindhwani S, Syed A, Ngai J, Kingston B, Maiorino L, Rothschild J (2020). The entry of nanoparticles into solid tumours. Nature materials.

[153] Bai S, Yang L, Wang Y, Zhang T, Fu L, Yang S (2020). Prodrug-Based Versatile Nanomedicine for Enhancing Cancer Immunotherapy by Increasing Immunogenic Cell Death. Small.

[154] Rios-Doria J, Durham N, Wetzel L, Rothstein R, Chesebrough J, Holoweckyj N (2015). Doxil synergizes with cancer immunotherapies to enhance antitumor responses in syngeneic mouse models. Neoplasia.

[155] Rao L, Wu L, Liu Z, Tian R, Yu G, Zhou Z (2020). Hybrid cellular membrane nanovesicles amplify macrophage immune responses against cancer recurrence and metastasis. Nat Commun.

[156] Zheng Y, Tang L, Mabardi L, Kumari S, Irvine D (2017). Enhancing Adoptive Cell Therapy of Cancer through Targeted Delivery of Small-Molecule Immunomodulators to Internalizing or Noninternalizing Receptors. ACS nano.

[157] Zhang Y, Li N, Suh H, Irvine D (2018). Nanoparticle anchoring targets immune agonists to tumors enabling anti-cancer immunity without systemic toxicity. Nat Commun.

[158] Ni Q, Zhang F, Liu Y, Wang Z, Yu G, Liang B (2020). A bi-adjuvant nanovaccine that potentiates immunogenicity of neoantigen for combination immunotherapy of colorectal cancer. Sci Adv.

[159] Han X, Shen S, Fan Q, Chen G, Archibong E, Dotti G (2019). Red blood cell-derived nanoerythrosome for antigen delivery with enhanced cancer immunotherapy. Sci Adv.

[160] Zhang Q, Wei W, Wang P, Zuo L, Li F, Xu J (2017). Biomimetic Magnetosomes as Versatile Artificial Antigen-Presenting Cells to Potentiate T-Cell-Based Anticancer Therapy. ACS nano.

[161] Chen Q, He Y, Wang Y, Li C, Zhang Y, Guo Q (2020). Penetrable Nanoplatform for "Cold" Tumor Immune Microenvironment Reeducation. Adv Sci.

[162] Sun Y, Zhang Y, Gao Y, Wang P, He G, Blum N (2020). Six Birds with One Stone: Versatile Nanoporphyrin for Single-Laser-Triggered Synergistic Phototheranostics and Robust Immune Activation. Adv Mater.

[163] Zhang N, Song J, Liu Y, Liu M, Zhang L, Sheng D (2019). Photothermal therapy mediated by phase-transformation nanoparticles facilitates delivery of anti-PD1 antibody and synergizes with antitumor immunotherapy for melanoma. J Control Release.

[164] Liu X, Zhang Y, Wang Y, Zhu W, Li G, Ma X (2020). Comprehensive understanding of magnetic hyperthermia for improving antitumor therapeutic efficacy. Theranostics.

[165] Zanganeh S, Hutter G, Spitler R, Lenkov O, Mahmoudi M, Shaw A (2016). Iron oxide nanoparticles inhibit tumour growth by inducing pro-inflammatory macrophage polarization in tumour tissues. Nat Nanotechnol.

[166] Liu X, Zheng J, Sun W, Zhao X, Li Y, Gong N (2019). Ferrimagnetic Vortex Nanoring-Mediated Mild Magnetic Hyperthermia Imparts Potent Immunological Effect for Treating Cancer Metastasis. ACS nano.

[167] Chavez M, Silvestrini M, Ingham E, Fite B, Mahakian L, Tam S (2018). Distinct immune signatures in directly treated and distant tumors result from TLR adjuvants and focal ablation. Theranostics.

[168] Ilovitsh T, Feng Y, Foiret J, Kheirolomoom A, Zhang H, Ingham E (2020). Low-frequency ultrasound-mediated cytokine transfection enhances T cell recruitment at local and distant tumor sites. Proc Natl Acad Sci U S A.

[169] Peske J, Thompson E, Gemta L, Baylis R, Fu Y, Engelhard V (2015). Effector lymphocyte-induced lymph node-like vasculature enables naive T-cell entry into tumours and enhanced anti-tumour immunity. Nat Commun.

[170] Cabrita R, Lauss M, Sanna A, Donia M, Skaarup Larsen M, Mitra S (2020). Tertiary lymphoid structures improve immunotherapy and survival in melanoma. Nature.

[171] Anandappa AJ, Wu CJ, Ott PA (2020). Directing Traffic: How to Effectively Drive T Cells into Tumors. Cancer Discov.

[172] Wu D, Wang S, Yu G, Chen X (2020). Cell Death Mediated by the Pyroptosis Pathway with the Aid of Nanotechnology: Prospects for Cancer Therapy. Angew Chem Int Ed.

[173] Li J, Anraku Y, Kataoka K (2020). Self-Boosting Catalytic Nanoreactors Integrated with Triggerable Crosslinking Membrane Networks for Initiation of Immunogenic Cell Death by Pyroptosis. Angew Chem Int Ed.

[174] Broz P, Pelegrín P, Shao F (2020). The gasdermins, a protein family executing cell death and inflammation. Nat Rev Immunol.

[175] Zhao P, Wang M, Chen M, Chen Z, Peng X, Zhou F (2020). Programming cell pyroptosis with biomimetic nanoparticles for solid tumor immunotherapy. Biomaterials.

[176] Cong Z, Zhang L, Ma S, Lam K, Yang F, Liao Y (2020). Size-Transformable Hyaluronan Stacked Self-Assembling Peptide Nanoparticles for Improved Transcellular Tumor Penetration and Photo-Chemo Combination Therapy. ACS nano.

[177] Zhang Y, Ma S, Liu X, Xu Y, Zhao J, Si X (2021). Supramolecular Assembled Programmable Nanomedicine As *In situ* Cancer Vaccine for Cancer Immunotherapy. Adv Mater.

